# Identification of the Metabolites of Both Formononetin in Rat Hepatic S9 and Ononin in Rat Urine Samples and Preliminary Network Pharmacology Evaluation of Their Main Metabolites

**DOI:** 10.3390/molecules28217451

**Published:** 2023-11-06

**Authors:** Yu-Zhu Yang, Tao Wang, Qi-Lei Chen, Hu-Biao Chen, Qian-Song He, Ya-Zhou Zhang

**Affiliations:** 1 College of Pharmacy, Guizhou University of Traditional Chinese Medicine, Guiyang 550025, China; 18385004834@163.com; 2 School of Chinese Medicine, Hong Kong Baptist University, Hong Kong SAR 999077, China; chenql@hkbu.edu.cn (Q.-L.C.); hubiao_chen@hkbu.edu.cn (H.-B.C.); 3 Departments of, Medicine and Biomedical Engineering, Faculty of Medicine and Health Sciences, McGill University, Montreal, QC H3G1Y6, Canada; wangtao201171@163.com; 4 First Clinical Medical College, Guizhou University of Traditional Chinese Medicine, Guiyang 550001, China

**Keywords:** hepatic S9, HPLC-DAD-ESI-IT-TOF-MS^n^, network pharmacology

## Abstract

*Astragalus membranaceus* is a traditional Chinese medicine derived from the roots of *Astragalus membranaceus* (Fisch.) Bge., which has the same medicinal and edible uses in China. It is also widely used in daily food, and its pharmacological effects mainly include antioxidant effects, vascular softening effects, etc. Currently, it is increasingly widely used in the prevention of hypertension, cerebral ischemia, and stroke in China. Formononetin and its glucopyranoside (ononin) are both important components of *Astragalus membranaceus*s and may play important roles in the treatment of cardiovascular diseases (CVDs). This study conducted metabolic studies using formononectin and its glucopyranoside (ononin), including a combination of the in vitro metabolism of Formonetin using rat liver S9 and the in vivo metabolism of ononin administered orally to rats. Five metabolites (Sm2, 7, 9, 10, and 12) were obtained from the solution incubated with formononetin and rat hepatic S9 fraction using chromatographic methods. The structures of the five metabolites were elucidated as (Sm2)6,7,4′-trihydroxy-isoflavonoid; (Sm7)7,4′-dihydroxy-isoflavonoid; (Sm9)7,8,4′-trihydroxy-isoflavonoid; (Sm10)7,8,-dihydroxy-4′-methoxy-isoflavonoid; and (Sm12)6,7-dihydroxy-4′-methoxy- isoflavonoid on the basis of UV, NMR, and MS data. Totally, 14 metabolites were identified via HPLC-DAD-ESI-IT-TOF-MS^n^ analysis, from which the formononetin was incubated with rat hepatic S9 fraction, and the main metabolic pathways were hydroxylation, demethylation, and glycosylation. Then, 21 metabolites were identified via HPLC-DAD-ESI-IT-TOF-MS^n^ analysis from the urine samples from SD rats to which ononin was orally administered, and the main metabolic pathways were glucuronidation, hydroxylation, demethylation, and sulfonation. The main difference between the in vitro metabolism of formononetin and the in vivo metabolism of ononin is that ononin undergoes deglycemic transformation into Formonetin in the rat intestine, while Formonetin is absorbed into the bloodstream for metabolism, and the metabolic products also produce combined metabolites during in vivo metabolism. The six metabolites obtained from the aforementioned separation indicate the primary forms of formononetin metabolism, and due to their higher contents of similar isoflavone metabolites, they are considered the main active compounds that are responsible for pharmacological effects. To investigate the metabolites of the active ingredients of formononetin in the rat liver S9 system, network pharmacology was used to evaluate the cardiovascular disease (CVD) activities of the six primary metabolites that were structurally identified. Additionally, the macromolecular docking results of six main components and two core targets (HSP90AA1 and SRC) related to CVD showed that formononetin and its main metabolites, Sm10 and Sm12, may have roles in CVD treatment due to their strong binding activities with the HSP90AA1 receptor, while the Sm7 metabolite may have a role in CVD treatment due to its strong binding activity with the SRC receptor.

## 1. Introduction

*Astragalus membranaceus* is a traditional Chinese medicine derived from the roots of *Astragalus membranaceus* (Fisch.) Bge., which has the same medicinal and edible uses in China. It is also widely used in daily food, and its pharmacological effects mainly include antioxidant effects, vascular softening effects, etc. Currently, it is increasingly widely used in the prevention of hypertension, cerebral ischemia, and stroke in China [1,2]. Formononetin and its glucopyranoside (ononin) are both important components of *Astragalus membranaceus*s, which may play crucial roles in treating cardiovascular diseases (CVDs) [3,4].

Formononetin, a widely distributed natural phytoestrogen, is an important active component in various traditional Chinese medicines, such as *Astragali radix*, *Ononis spinosa*, *Trifolium pratense* L., etc. Its effectiveness has been demonstrated in cancer prevention, in cardiovascular function protection, and in osteoporosis treatment [1,2,3,4,5].

Ononin, the 7-*O*-glucoside of formononetin, is another significant active constituent and severs as the precursor of formononetin in plants. It exhibits diverse biological activities, including immune regulation, antioxidant effects, and phytoestrogenic properties [6,7,8].

Previous studies on the metabolism of formononetin have indicated numerous metabolites and metabolic pathways, encompassing a 2,3-double bond reduction (dihydroformononetin), 4-carbonyl reduction (equol), open-loop of the B-ring (angolensin), hydroxylation and demethylation reactions, and all metabolites identified through a high-resolution spectral analysis [9,10,11,12,13]. Therefore, it is necessary to investigate the metabolic process of ononin and compare it to that of formononetin by examining the differences in their internal and external metabolisms. By comparison, formononetin was used in rat liver S9 for in vitro metabolic studies to elucidate the main metabolic functions of the liver; however, the overall administration of ononin involves other possible metabolites besides liver metabolism, such as the effects of the gutbacteria and kidneys. The impact of these metabolites with the same isoflavone structure on cardiovascular disease remains unstudied.

Due to the limited oral absorption of formononetin and ononin, it was challenging to comprehensively elucidate the structures of their metabolites though the entire experiment covering the whole metabolic process [14,15]. When administered via oral gavage, ononin is metabolized into formononetin and then absorbed into the blood in the rat intestine [16,17]. This study aims to profile the main metabolites of formononetin in vitro and the main metabolites of ononin in vivo. The formononetin was incubated in hepatic S9 in vitro (mainly phase Ⅰ metabolites) and the ononin was administered via oral gavage to rats in vivo (mainly phase Ⅱ metabolites) [14,15,18,19]. The metabolites in the above samples were analyzed using high-resolution mass spectrometry (HPLC-DAD-ESI-IT-TOF-MS^n^), and for the rat liver S9 experimental sample of formononetin, we isolated the major metabolites using chromatographic techniques. Due to the fact that these metabolites share the same isoflavone structure, the higher their contents, the more likely they are to be main active forms for treating associated diseases. Furthermore, the isoflavones in *Astragalus membranaceus* have long been recognized in traditional Chinese medicine for the treatment of CVDs [1,2]. Finally, network pharmacology was employed to study the core metabolites and potential targets associated with CVDs, aiming to identify their main receptors involved in the treatment of CVDs [20,21,22,23]. Moreover, a comparative analysis of the binding activities of these metabolites with those of the respective receptors was conducted, aiming to identify the metabolite exhibiting the highest binding affinity towards the corresponding receptor. Such findings will guide the future applications of formononetin, onion, and their main metabolites [24].

## 2. Results and Discussion

### 2.1. Isolation and Determination of the Structure of Formononetin Metabolites

By means of chromatographic methods, five metabolites (Sm2, 7, 9, 10, and 12) were isolated from rat hepatic S9 mixtures incubated with formononetin (Table 1).

Compound Sm2

Sm2 was obtained as a white powder, and it showed [M+H]^+^ at *m*/*z* 271.0597 (predicted to be C_15_H_11_O_5_; theoretical mass: 271.0606; error: −3.32 ppm) in the PI (positive ionization) mass spectrum of HR-TOF-MS, indicating that its molecular formula was C_15_H_10_O_5_. The molecular formula had one more oxygen atom and one less methylene than that of formononetin, suggesting that Sm2 is a monohydroxylated and demethylated metabolite.

^1^H-NMR and ^13^C-NMR data: In the ^1^H-NMR spectrum, the signal assigned to H-OCH_3_ was not observed at δ 3.77 (3H, s) unlike that of formononetin, while it showed one more hydroxyl at δ 9.74 (1H, brs). The signal assigned to H-6 was not observed at δ 6.91 (1H, d, *J* = 2.0, 8.8 Hz), unlike that of formononetin, while it showed one more hydroxyl at δ 9.48 (1H, brs). The ^13^C-NMR spectrum of Sm2 revealed 15 carbon signals and had one less methyl signal at δ 55.6 (C-OCH_3_) in contrast to that of formononetin, suggesting that demethylation occurred at the methoxyl group linked to C-4′. Sm2 exhibited a C-6 signal at δ 152.6, which was shifted downfield of that of C-8 at δ 115.6 in formononetin, suggesting that the newly added hydroxyl group was linked to C-6. The structure of Sm2 was found to be as 6-hydroxy-4′-demethyl-formononetin via a comparison of its spectral data to those of 6-hydroxy-4′-demethyl-formononetin in the literature [9].

Compound Sm7

Sm7 was obtained as a white powder, and it showed [M+H]^+^ at *m*/*z* 255.0659 (predicted to be C_15_H_11_O_4_; theoretical mass: 255.0657; error: 0.78 ppm) in the PI mass spectrum of HR-TOF-MS, indicating that its molecular formula was C_15_H_10_O_4_. The molecular formula had one less methylene than that of formononetin, suggesting that Sm7 was a demethylated metabolite.

^1^H-NMR and ^13^C-NMR data: In the ^1^H-NMR spectrum, the signal assigned to H-OCH_3_ was not observed at δ 3.77 (3H, s) in contrast to that of formononetin, and it showed one more hydroxyl at δ 9.50 (1H, brs). The ^13^C-NMR spectrum of Sm7 revealed 15 carbon signals, and it had one less methyl signal at δ 55.6 (C-OCH_3_), unlike that of formononetin, suggesting that the demethylation occurred at the methoxyl group linked to C-4′. The structure of Sm7 was elucidated as 4′-demethyl-formononetin (daizein) via a comparison of its spectral data to those of daizein in the literature [25].

Compound Sm9

Sm9 was obtained as a white powder, and it showed [M+H]^+^ at *m*/*z* 271.0597 (predicted to be C_15_H_11_O_5_; theoretical mass: 271.0606; error: −3.32 ppm) in the PI mass spectrum of HR-TOF-MS, indicating that its molecular formula was C_15_H_10_O_5_. The molecular formula had one more oxygen atom and one less methylene atom than that of formononetin, suggesting that Sm9 was a monohydroxylated and demethylated metabolite.

^1^H-NMR and ^13^C-NMR data: In the ^1^H-NMR spectrum, the signal assigned to H-OCH_3_ was not observed at δ 3.77 (3H, s) in contrast to that of formononetin, and it showed one more hydroxyl at δ 9.50 (1H, brs), and the signal assigned to H-8 was not observed at δ 6.85 (1H, d, *J* = 2.0 Hz) in contrast to that of formononetin, and showed one more hydroxyl at δ 9.40 (1H, brs). The ^13^C-NMR spectrum of Sm9 revealed 15 carbon signals, and it had one less methyl signal at δ 55.6 (C-OCH_3_), unlike formononetin. This suggests that demethylation occurred at the methoxyl group linked to C-4′.

Sm9 exhibited a C-8 signal at δ 133.3, which was shifted downfield relative to that of C-8 at δ 102.6 in formononetin, suggesting that the newly added hydroxyl group was linked to C-8. The structure of Sm9 was found to be 8-hydroxy-4′-demethyl-formononetin via a comparison of its spectral data to those of 8-hydroxy-4′-demethyl-formononetin in the literature [26].

Compound Sm10

Sm10 was obtained as a white powder, and it showed [M+H]^+^ at *m*/*z* 285.0755 (predicted to be C_16_H_13_O_5_; theoretical mass: 285.0763; error: −2.81 ppm) in the PI mass spectrum of HR-TOF-MS, indicating that its molecular formula was C_16_H_12_O_5_. The molecular formula had one more oxygen atom than that of formononetin, suggesting that Sm10 was a monohydroxylated metabolite.

^1^H-NMR and ^13^C-NMR data: In the ^1^H-NMR spectrum, the signal assigned to H-8 was not observed at δ 6.85 (1H, d, *J* = 2.0 Hz), unlike that of formononetin. One more hydroxy was observed at δ 9.42 (1H, brs). The ^13^C-NMR spectrum of Sm10 exhibited a C-8 signal at δ 133.4, which was shifted downfield relative to that of C-8 at δ 102.6 in formononetin, suggesting that the newly added hydroxyl group was linked to C-8. In this way, the structure of Sm10 was found to be 8-hydroxy-formononetin via a comparison of its spectral data to those of 8-hydroxy-formononetin in the literature [27].

Compound Sm12

Sm12 was obtained as a white powder, and it showed [M+H]^+^ at *m*/*z* 285.0811 (predicted to be C_16_H_13_O_5_; theoretical mass: 285.0763; error: −1.77 ppm) in the PI mass spectrum of HR-TOF-MS, indicating that its molecular formula was C_16_H_12_O_5_. The molecular formula had one more oxygen atom than that of formononetin, suggesting that Sm12 was a monohydroxylated metabolite.

^1^H-NMR and ^13^C-NMR data: In the ^1^H-NMR spectrum, the signal assigned to H-6 was not observed at δ 7.94 (1H, d, *J* = 8.8 Hz), unlike that of formononetin, and it showed one more hydroxyl at δ 9.77 (1H, brs). The ^13^C-NMR spectrum of Sm12 exhibited a C-6 signal at δ 152.7, which was shifted downfield relative to that of C-6 at δ 115.6 in formononetin, suggesting that the newly added hydroxyl group was linked to C-6. In this way, the structure of Sm12 was found to be 6-hydroxy-formononetin via a comparison of its spectral data to those of 6-hydroxy-formononetin in the literature [28].

### 2.2. MS^n^ Fragmentation Behavior of Formononetin

**Figure 1:**
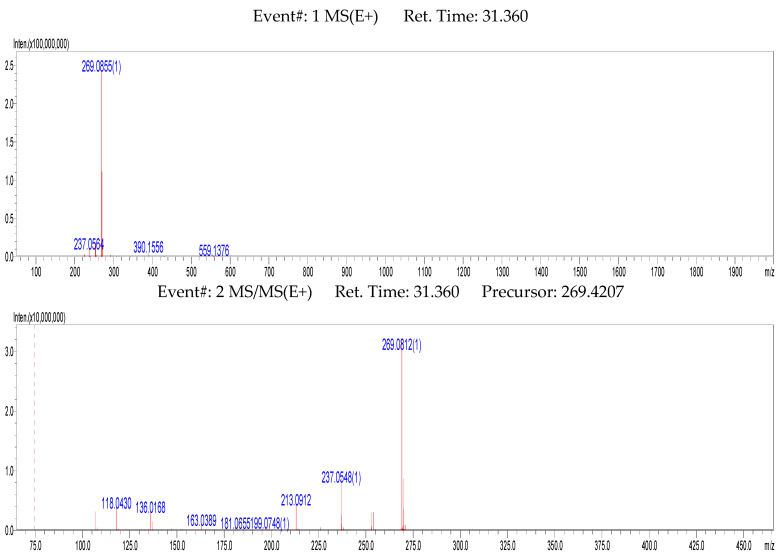

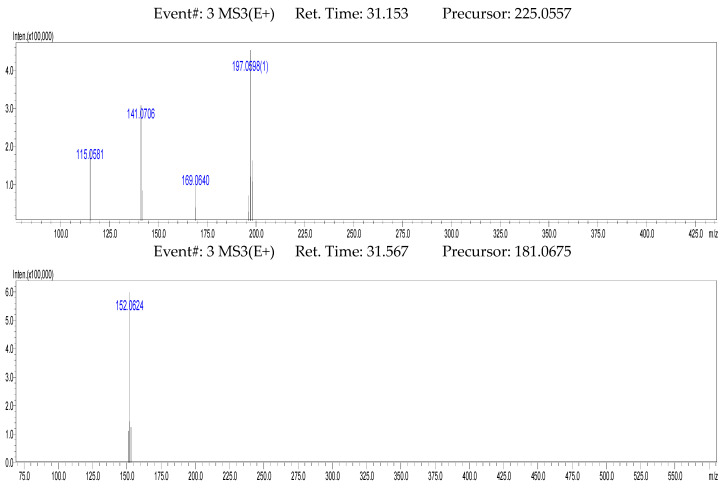
MS^1–3^ data of formononetin in positive ion (PI) LC/MS spectrum.

To identify and help elucidate the structures of the metabolites, we first studied the MS^n^ fragmentation behavior of formononetin in the PI (positive ionization) mode and NI (negative ionization) mode. We found that the fragmentation behavior in the PI mode can give more information about the structure than in the NI mode [29,30,31]. We elucidated the structure of the metabolites mostly via a PI mass spectrum, which is shown in Figure 1.

**Figure 2:**
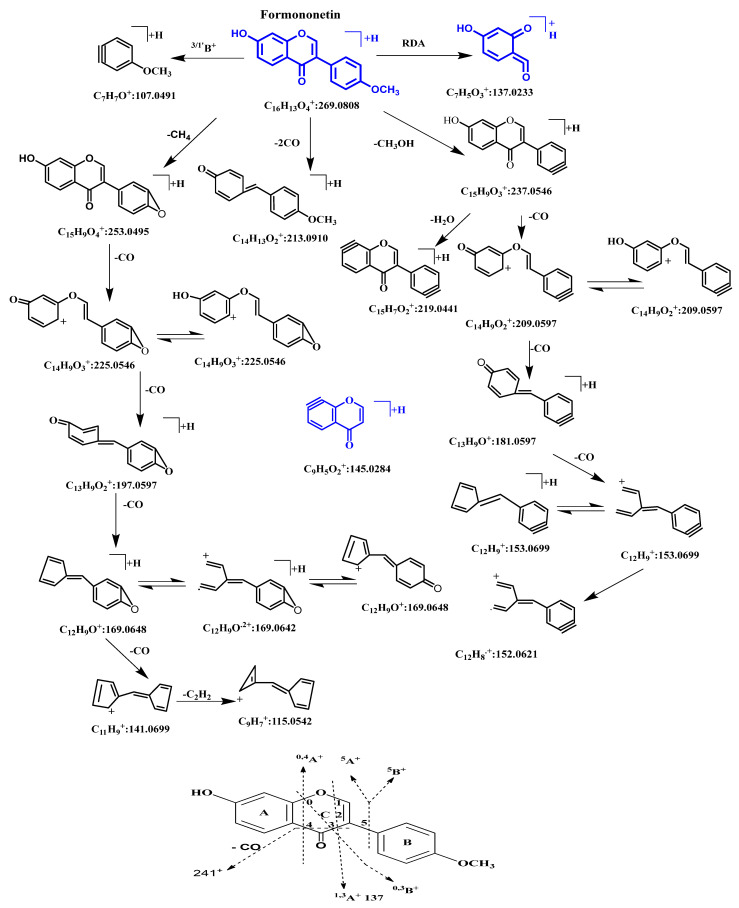
Proposed major fragmentation pathways and nomenclature adopted for cross-ring cleavages in PI mode of formononetin.

Formononetin had a retention time of 31.205 min. It showed [M+H]^+^ at *m*/*z* 269.0852 (predicated mass: 269.0773; error: 4.49 ppm; element composition: C_16_H_13_O_4_) in the PI mass spectrum and showed [M−H]^−^ at *m*/*z* 267.0669 in the NI mass spectrum. Its molecular formula was determined to be C_16_H_12_O_4_. In the MS^2^ PI mass spectrum, it showed a base peak at *m*/*z* 269.0812 [M+H]^+^ and was accompanied by several distinctive fragment ions, including ^5^B^+^–2H, ^5^A^+^–2H, ^1,3^A^+^–2H, M^+^–CH_3_OH, M^+^–CH_3_OH–CO, M^+^–CH_4_, etc. [29,30,31,32,33]. Based on the MS^2^ and MS^3^ spectra, its major fragmentation pathways in the PI mode were proposed and are shown in Figure 2.

We used the characteristic retro-Diels–Alder (RDA) cracking fragment (^1,3^A^+^–2H; 137) of isoflavone to determine the oxidation of the metabolites; the glucose- and pentose-binding metabolites, with a neutral loss (−162; 132) of the molecular ion peak in the MS^2^ spectra; glucuronidated metabolites with a neutral loss (−176) of the molecular ion peak in the MS^2^ spectra; and sulfated metabolites with a neutral loss (−80) of the molecular ion peak in the MS^2^ spectra.

### 2.3. Metabolite Profiling of Formononetin Using Rat Hepatic S9 by HPLC-DAD-ESI-IT-TOF-MS^n^ Analysis

**Figure 3:**
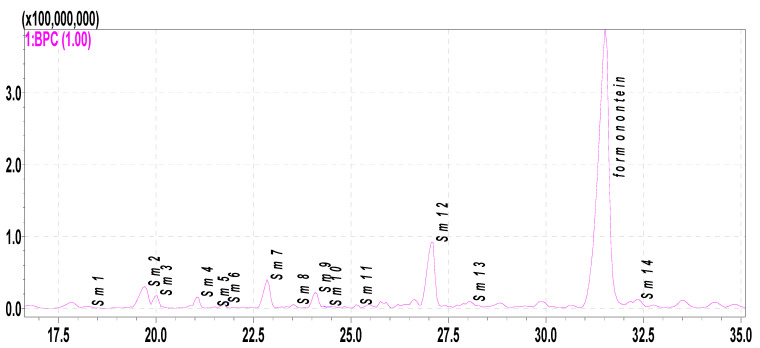
Proposed metabolites of formononetin incubated in hepatic S9 as indicated via LC/MS analysis (BPC).

**Figure 4:**
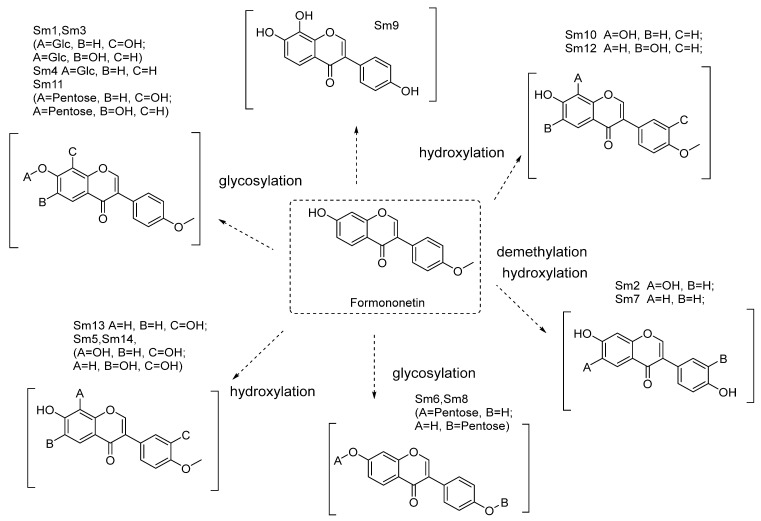
Proposed metabolites of formononetin in hepatic S9 incubated samples.

The metabolites of formononetin in the rat hepatic S9 fraction were analyzed through HPLC-DAD-ESI-IT-TOF-MS^n^. In this research, 14 metabolites were found and tentatively identified (Figure 3 and Figure 4 and Table 2). Their MS data are shown in the Supplementary Materials (pages 2–9).

S0 was identified as the prototype of formononetin.

Metabolites (glycosylation)—Sm1, Sm3, Sm4, Sm6, Sm8, Sm9, and Sm11

Based on the exact masses of the ions, all of these metabolites had a neutral loss (−162; 132) of the molecular ion peak in the MS^2^ spectra, suggesting that they were glycosylation metabolites of formononetin.

Sm6, Sm8, and Sm11 showed [M+H]^+^ at *m*/*z* 417.12 (element composition: C_21_H_21_O_9_) in the PI mass spectrum. So, the molecular formula was determined to be C_21_H_20_O_9_ with a neutral loss (132) of the molecular ion peak in the MS^2^ spectra, thus suggesting that they were hydroxylation and glycosylation (pentose) metabolites of formononetin.

Sm4 showed [M+H]^+^ at *m*/*z* 431.13 (element composition: C_22_H_23_O_9_) in the PI mass spectrum. So, the molecular formula was determined to be C_22_H_22_O_9_ with a neutral loss (162) of the molecular ion peak in the MS^2^ spectra. Thus, those observations suggested that Sm4 was a glycosylation (glycose) metabolite of formononetin.

Sm1 and Sm3 showed [M−H]^−^ at *m*/*z* 445.11 (element composition: C_22_H_23_O_10_) in the PI mass spectrum. So, their molecular formula was determined to be C_22_H_22_O_10_ with a neutral loss (162) of the molecular ion peak in the MS^2^ spectra. The above information suggests that they were hydrolation and glycosylation (glycose) metabolites of formononetin.

Metabolites (hydroxylation)—Sm10, Sm12, Sm13, and Sm5

Sm10, Sm12, and Sm13 showed [M+H]^+^ at *m*/*z* 285.08 (element composition: C_16_H_13_O_5_) in the PI mass spectrum. So, their molecular formula were determined to be C_16_H_12_O_5_, thus suggesting that they were hydroxylation metabolites of formononetin. Sm10 was 7,8,-dihydroxy-4′-methoxy-isoflavonoid and Sm12 was 6,7-dihydroxy-4′-methoxy- isoflavonoid.

Sm5 showed [M+H]^+^ at *m*/*z* 301.07 (element composition: C_16_H_13_O_6_) in the PI mass spectrum. So, the molecular formula was determined to be C_16_H_12_O_6_, thus suggesting that it was a dihydroxylation metabolite of formononetin.

Metabolites (demethylation, methylation, and hydroxylation)—Sm7, Sm14, and Sm2

Sm7 showed [M+H]^+^ at *m*/*z* 255.07 (element composition: C_15_H_11_O_4_) in the PI mass spectrum. So, the molecular formula was determined to be C_15_H_10_O_4_, thus suggesting that Sm7 was a demethylation metabolite of formononetin. Sm7 was 7,4′-dihydroxy-isoflavonoid.

Sm14 showed [M+H]^+^ at *m*/*z* 299.39 (element composition: C_17_H_15_O_5_) in the PI mass spectrum. So, the molecular formula was determined to be C_17_H_14_O_5_, thus suggesting that Sm14 was a methylation and hydroxylation metabolite of formononetin.

Sm2 and Sm9, showed [M+H]^+^ at *m*/*z* 271.06 (element composition: C_15_H_11_O_5_) in the PI mass spectrum. So, the molecular formula was determined to be C_15_H_10_O_5_, thus suggesting that Sm2 was a demethylation and hydroxylation metabolite of formononetin. Sm2 was 6,7,4′-trihydroxy-isoflavonoid, and Sm9 was a demethylation and hydroxylation metabolite of formononetin. Sm9 was 7, 8, 4′-trihydroxy-isoflavonoid.

### 2.4. Metabolite Profiling of Rat Urine Samples after Oral Administration of Ononin via HPLC-DAD-ESI-IT-TOF-MS^n^ Analysis

**Figure 5:**
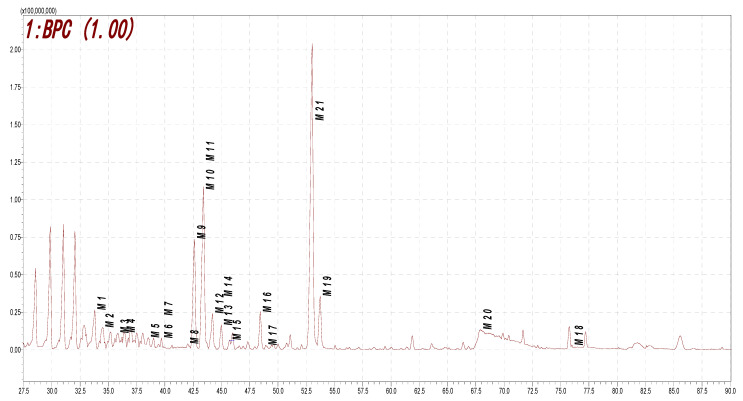
Proposed metabolites of ononin in rat biological sample as indicated via LC/MS analysis (BPC).

**Figure 6:**
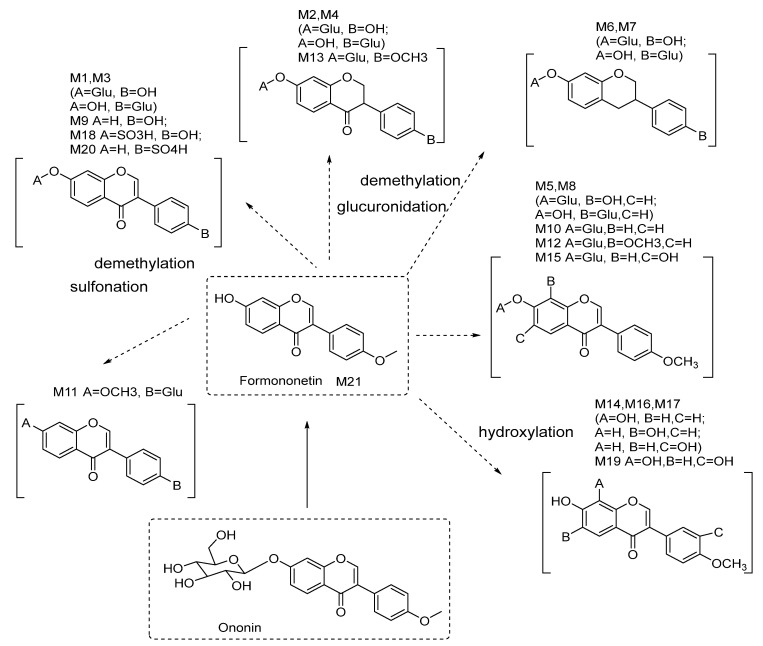
Proposed metabolites of ononin in rat biological samples as indicated via LC/MS analysis.

The metabolites in the rat urine samples collected after the oral administration of ononin were analyzed using the HPLC-DAD-ESI-IT-TOF-MS^n^ technique. In total, 21 metabolites were found and tentatively identified, as shown in Figure 5 and Figure 6 and in Table 3. Their MS data are shown in the Supplementary Materials (pages 11–17).

Metabolites (glucuronidation)—M1, M2, M3, M4, M5, M6, M7, M8, M10, M11, M12, M13, and M15

Based on the exact masses of the ions, all of these metabolites had a neutral loss (−176) of the molecular ion peak in the MS^2^ spectra, suggesting that they were glucuronidation metabolites of formononetin.

M10 and M11 showed [M+H]^+^ at *m*/*z* 445.11 (element composition: C_22_H_21_O_10_) in the PI mass spectrum. So, the molecular formula was determined to be C_22_H_20_O_10_ with a neutral loss (176) of the molecular ion peak in the MS^2^ spectra, thus suggesting that they were glucuronidation metabolites of formononetin.

M1 and M3 showed [M−H]^−^ at *m*/*z* 429.08 (element composition: C_21_H_19_O_10_) in the PI mass spectrum. So, the molecular formula was determined to be C_21_H_18_O_10_ with a neutral loss (176) of the molecular ion peak in the MS^2^ spectra, thus suggesting that they were demethylation and glucuronidation metabolites of formononetin.

M5, M8, and M15 showed [M−H]^−^ at *m*/*z* 459.09 (element composition: C_22_H_21_O_11_) in the PI mass spectrum. So, the molecular formula was determined to be C_22_H_20_O_11_ with a neutral loss (176) of the molecular ion peak in the MS^2^ spectra, thus suggesting that they were hydroxylation and glucuronidation metabolites of formononetin.

M6 and M7 showed [M−H]^−^ at *m*/*z* 417.12 (element composition: C_21_H_23_O_9_) in the PI mass spectrum. So, the molecular formula was determined to be C_21_H_22_O_9_ with a neutral loss (176) of the molecular ion peak in the MS^2^ spectra, thus suggesting that they were hydrogenation, carbonyl reduction, and glucuronidation metabolites of formononetin.

M2 showed [M−H]^−^ at *m*/*z* 431.10 (element composition: C_21_H_21_O_10_) in the PI mass spectrum. So, the molecular formula was determined to be C_21_H_20_O_10_ with a neutral loss (176) of the molecular ion peak in the MS^2^ spectra, thus suggesting that M2 was a hydrogenation, demethylation, and glucuronidation metabolite of formononetin.

M12 showed [M+H]^+^ at *m*/*z* 475.12 (element composition: C_23_H_23_O_11_) in the PI mass spectrum. So, the molecular formula was determined to be C_23_H_22_O_11_ with a neutral loss (176) of the molecular ion peak in the MS^2^ spectra, thus suggesting that M12 was a hydroxylation, methylation, and glucuronidation metabolite of formononetin.

M13 showed [M+H]^+^ at *m*/*z* 447.13 (element composition: C_22_H_23_O_10_) in the PI mass spectrum. So, the molecular formula was determined to be C_22_H_22_O_10_ with a neutral loss (176) of the molecular ion peak in the MS^2^ spectra, thus suggesting that M13 was a hydrogenation, demethylation, and glucuronidation metabolite of formononetin.

Metabolites (sulfonation)—M19 and M20

Based on the exact masses of the ions, all of these metabolites had a neutral loss (−80) of the molecular ion peak in the MS^2^ spectra, suggesting that they were sulfonation metabolites of formononetin.

M19 and M20 showed [M+H]^+^ at *m*/*z* 335.02 (element composition: C_15_H_11_O_7_S) in the PI mass spectrum. So, the molecular formula was determined to be C_15_H_10_O_7_S with a neutral loss (80) of the molecular ion peak in the MS^2^ spectra, thus suggesting that they were demethylation and sulfonation metabolites of formononetin.

Metabolites (hydroxylation, demethylation, and methylation)—M9, M14, M16, M17, and M18

M14, M16, and M17 showed [M+H]^+^ at *m*/*z* 285.08 (element composition: C_16_H_13_O_5_) in the PI mass spectrum. So, the molecular formula was determined to be C_16_H_12_O_5_, thus suggesting that they were hydroxylation metabolites of formononetin.

M9 showed [M+H]^+^ at *m*/*z* 255.07 (element composition: C_15_H_11_O_4_) in the PI mass spectrum. So, the molecular formula was determined to be C_15_H_10_O_4_, thus suggesting that M9 was a demethylation metabolite of formononetin.

M18 showed [M+H]^+^ at *m*/z 299.09 (element composition: C_17_H_15_O_5_) in the PI mass spectrum. So, the molecular formula was determined to be C_17_H_14_O_5_, thus suggesting that M18 was a hydroxylation and methylation metabolite of formononetin.

M21 showed [M+H]^+^ at *m*/*z* 269.08 (element composition: C_16_H_13_O_4_) in the PI mass spectrum. So, the molecular formula was determined to be C_16_H_12_O_4_, thus suggesting that M18 was the formononetin.

### 2.5. Results of Network Pharmacology and Macromolecular Docking

**Figure 7:**
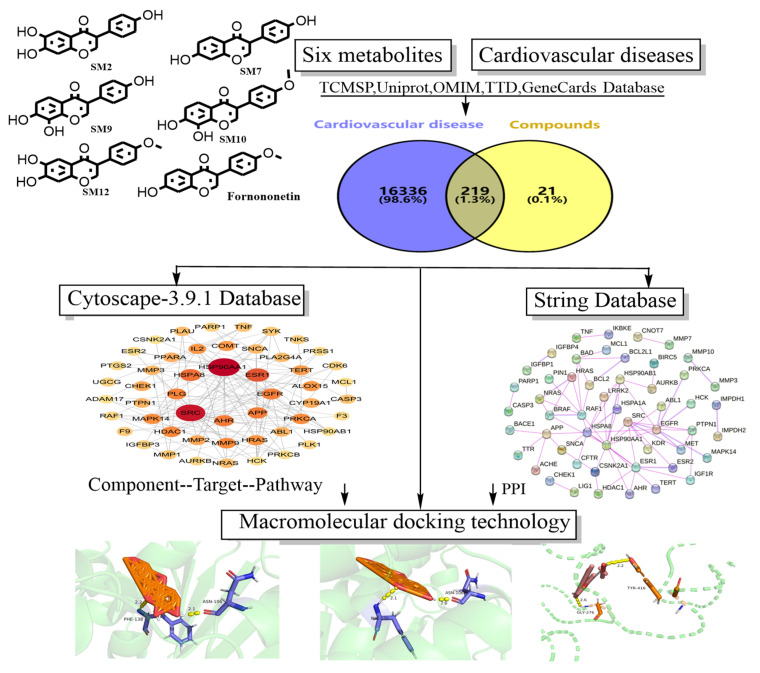
Schematic diagram of network pharmacology and macromolecular docking of six separated metabolites.

A search for targets corresponding to the six metabolites was conducted, the duplicates were deleted, and a total of 240 targets were obtained. Through the Genecards database (https://auth.lifemapsc.com/), 16,554 cardiovascular disease prediction targets were obtained. The active ingredient cardiovascular disease target was venny 2.1.0., 219 intersection targets were obtained, and the intersection rate was 1.3%. The intersection targets were submitted in the String11.0 database to obtain the intersection target protein interaction network diagram. The topological map of the intersection target of the active ingredient and cardiovascular disease was exported based on the Betweenness Centrality value in the Cytoscape 3.9.1 software. Ranked by the Betweenness Centrality value, the first two core targets (HSP90AA1 and SRC) related to cardiovascular disease were selected for macromolecular docking with six core components, and the results are shown in Figure 7 and Table 4 and Table 5.

The macromolecular docking results of the six main components and two core targets (HSP90AA1 and SRC) related to cardiovascular diseases showed that the binding energies of Sm2, Sm9, Sm12, and formononetin with the SRC target and the binding energies of the Sm7 components with the HSP90AA1 target were all less than −4.25 kcal/mol. This indicates that there is certain activity between Sm7 and the HSP90AA1 receptor, and there is certain activity between Sm2, Sm9, Sm12, and formononetin with the SRC receptor of cardiovascular disease; however, the binding energies of Sm9, Sm12, and formononetin with the HSP90AA1 target, and the binding energy of Sm7 with the SRC target are all less than −5.0 kcal/mol, indicating that Sm10, Sm12, and formononetin have good binding activities with HSP90AA1 receptors, and there is also a good binding activity between Sm7 with the SRC receptors.

### 2.6. Discussion

We evaluated the major fragmentation pathways of formononetin and its metabolites, which were identified using the NMR methods, and then speculated on the proposed metabolites.

Fourteen metabolites were identified when formononetin was incubated with the rat hepatic S9 fraction. Additionally, when ononin was administered orally to the rats, 21 previously unknown metabolites were simultaneously identified in their urine through an HPLC-DAD-ESI-IT-TOF-MS^n^ analysis [1,14,15,16].

The research on the metabolic profiling analysis referred to previous studies in the literature to identify the structures of the metabolites [9,10,11,12,13]. When formononetin was incubated in hepatic S9 and the ononin was administered orally to the rats, these metabolic pathways were involved in hydroxylation and demethylation. These results indicate that hydroxylation and demethylation are the main phase I metabolic reactions in the hepatic transformation of formononetin in vitro and in the metabolism of ononin in vivo, and it was also confirmed that hydroxylation occurred most at carbon-6 and carbon-8 of the A-ring based on the NMR data [10,11]. The combination reaction when formononetin was incubated in hepatic S9 was mainly glycosylation; however, when ononin was administered via oral gavage to the rats, the combination reactions were mainly glucuronidation and sufonation, and a glycosylation reaction in rabbit liver microsomal fraction and zebrafish larvae been reported, too [34,35,36]. The present study is also the first to report that formononetin is able to combine with glucose (or pentose) when incubated in rat hepatic S9, but a reaction was not observed when ononin was orally administrated in vivo. Compared with formononetin incubated with rat hepatic S9 fraction, we found that the open-loop reaction of the B-ring, the 2,3-double bond reduction, and the 4-carbonyl reduction were transformed by the intestinal bacteria during the metabolism of ononin before it was absorbed into the blood [11,12,13].

When we study the metabolism of ononin, because there is poor absorption through oral administration in vivo, it is difficult to isolate and identify each metabolite [14,15]. However, the S9 fraction can be obtained via a simple modification to the crude S9 preparation and more accurately reflects the metabolism as a metabolic system; it was much easier to apply hepatic S9 incubation to the isolation and structural identification of those metabolites. This was the first time to describe the metabolic profiling of ononin by using rat liver S9 incubation in vitro in combination with administrated by oral gavage in vivo. 

Finally, the researchers conducted a network pharmacological analysis of the main components in similar structures, hoping to provide a reference for the subsequent determination of active structures. Through network pharmacology and macromolecular docking, the relationship between six metabolic components isolated from rat liver S9 after the hatching of formononetin and potential targets of CVD were analyzed, and six metabolites were found to be involved in the main roles of 10 receptors in the treatment of CVD. We compared the binding activity of these metabolites with that of the first two receptors (HSP90AA and SRC) and found the metabolites with the strongest binding abilities to the corresponding receptors. These research results also indicate that a prototype drug may produce a large number of metabolites after administration, and the specific molecular form that plays a therapeutic role may be the prototype or may be metabolites produced by the prototype, which require a specific analysis. In summary, the above research on formononetin and its glycosides (onion) has a certain guiding role for their further applications [22,23].

Because the content of isoflavone glycoside is generally greater than that of aglycone in plants, when understanding the metabolites of both formononetin and ononin, it is helpful to evaluate their safety after they enter the body [1,3,4]. It has been demonstrated that some of the metabolites of both formononetin and ononin have shown effective activities according to previous pharmacological studies [37,38,39]. The findings in this research will provide a solid basis for further studies on the metabolism of other isoflavonoids in animals, too.

## 3. Materials and Methods

### 3.1. Materials and Reagents

Formononetin and ononin (both >98%) were isolated from *Astragali Radix* (the roots of *Astragalus membranaceus* (Fish.) Bge. ), which was identified by Ya-Zhou Zhang using chromatographic methods, including column chromatography on D-101 macroporous adsorption resin (Cangzhou Baoen Co., Ltd., Cangzhou, Hebei Province, China), 200–300 mesh normal-phase silica gel (Qingdao Marine Chemical Factory, Qingdao, Shandong Province, China), reversed-phase C_18_ silica gel (YMC, YMC Co., Ltd., Kyoto, Japan), and Sephadex LH-20 (GE Healthcare Co., Ltd., Chicago, IL, USA). The structures were determined to be formononetin and ononin on the basis of UV, NMR, and MS data. HPLC-grade acetonitrile was purchased from Fisher Chemicals (Princeton, NJ, USA), pure water was purchased from Wahaha Co., Ltd. (Hangzhou, Zhejiang Province, China) and NADP, glucose-6-phosphate, and Tris-HCl buffer were purchased form Sigma, Co., Ltd. (St. Louis, MO, USA). Other reagents (sodium chloride, potassium hydroxide, hydrochloride, and dimethyl sulfoxide) were of analytical grade and were purchased from Beijing Fine Chemicals Co., Ltd. (Beijing, China).

### 3.2. Ethical Approval of Animal Experiments

Adult male Sprague Dawley rats (250–350 g) were bought from the Experimental Animal Center of Guizhou University of Traditional Chinese Medicine. They were handled in accordance with the Guide for the Care and Use of Laboratory Animals of the USA’s National Institutes of Health. The experiments were also approved by the Biomedical Ethical Committee of Guizhou University of Traditional Chinese Medicine (No. 776).

### 3.3. Preparation of the Rat Hepatic S9 Fraction

The rats were induced via i.p. phenobarbital with a dosage of 60 mg/kg before use, the rats were euthanized via decapitation 24 h later, and the livers were removed from the abdominal cavities quickly, and then their wet weights were determined. Subsequently, the livers were minced and homogenized using a homogenizer (Ultra-Turrax T8, Ika-Werke, Gmbh & Co. KG, Staufen, Germany) in cold 1.15% KCl in 0.05 M Tris-HCl buffer (pH 7.4) to the 4-fold volume of the wet liver weight. The homogenate was centrifuged at 9000× *g* for 30 min at 4 °C. The 9000× *g* supernatant (S9) was removed and either stored at −70 °C or used immediately [18,19].

### 3.4. Rat Hepatic S9 Incubation for Metabolite Profiling

The general procedure of incubation was conducted as previously reported [18]. In brief, 25 mL each of 1.15% KCl, 5 mM MgCl_2_, 5 mM glucose-6-phosphate, 0.5 mM NADP, and hepatic S9 fraction (20 mg protein per mL) in 0.05 M Tris-HCl buffer (pH 7.4) were successively added to a 250 mL flask (on ice) to obtain a final volume of 125 mL. The incubation was conducted in three groups: (1) A test group for metabolite profiling was incubated with 100 mL cofactors and 25 mL hepatic S9 and spiked with 10 mg formononetin (100 μL DMSO solution). (2) Control group 1, excluding endogenous S9 metabolites, was incubated with 100 mL cofactors and 25 mL hepatic S9 but no formononetin. (3) Control group 2, to evaluate the stability of formononetin, was incubated with 125 mL cofactors (25 mL hepatic S9 was replaced by 25 mL 1.15% KCl in Tris-HCl) spiked with 10 mg formononetin (100 μL DMSO solution).

### 3.5. Enlarged Rat Hepatic S9 Incubation for Isolation of Metabolites

The incubation method was the same as that described in previous reports [18]. In total, 400 mg formononetin was dissolved in 2.4 mL DMSO and added to 5000 mL reaction mixture (containing 1000 mL rat hepatic S9 fraction). Then, the mixture was incubated at 37 °C (each 125 mL for incubation). After 4 h, 5000 mL acetonitrile was added to completely stop the reaction. The mixture was centrifuged for 30 min (200 or 300 mL per round of centrifugation), and the supernatant was evaporated under reduced pressure to produce a solution (200 mL). All the solutions were subjected to D-101 macroporous adsorption resin column chromatography. They were first eluted with water and then with methanol to give two fractions. Metabolite 1 (10.8 mg), metabolite 2 (11.1 mg), metabolite 3 (10.2 mg), metabolite 4 (2.3 mg), and metabolite 5 (4.2 mg) were isolated from fraction 2. Then, each metabolite was purified on a preparative HPLC system (including an Alltech 426 HPLC pump and a UVIS 200 detector). The purity of each metabolite was found to be above 98% via HPLC analysis using an Agilent 1200 series instrument equipped with a binary gradient system, a thermostatted column oven, and a DAD-UV detector.

### 3.6. Rats’ Urine Samples Collected after Oral Administered with Ononin

Male SD rats (250–350 g, *n* = 3) were given ononin via oral gavage at a dose of 400 mg/kg body weight. We collected the urine 48 h later and labeled it sampling A. Urine collected 48 h before administration were labeled blank B. Urine was concentrated to dryness at 40 °C and the precipitate was dissolved with methanol and 30 mL supersonic for 10 min. After that, supernatant was accepted and filtered through the millipore filter (0.45 μm) before HPLC-DAD-ESI-IT-TOF-MS^n^ analysis.

### 3.7. The Instrument Parameters of HPLC-DAD-ESI-IT-TOF-MS^n^

The analyses were performed on a Shimadzu liquid phase chromatography and mass spectrograph (LC/MS) tandem with an IT-TOF instrument, which consisted of a CBM-20A system controller, two LC-20AD pumps, an SIL-20AC autosampler, a CTO-20A column oven, an SPD-M20A PDA detector, an ESI ion source, and an IT-TOF mass spectrometer.

The ESI-IT-TOF-MS^n^ parameters were set as follows: (1) Flow rate: 0.2000 mL/min (split from HPLC effluent). (2) Detected in alternating positive ion (PI) and negative ion (NI) mode. (3) Ion source temperature: 250 °C; curved desolvation line temperature: 250 °C; ESI nebulization gas flow (nitrogen): 1.5 L/min; ESI voltage: 4.5 kV; detector voltage: 1.80 kV; ion accumulation time: 20 ms; relative collision energy: 50%. (4) Mass range: *m*/*z* 220–1000 in MS, *m*/*z* 50–1000 in MS^2^ and MS^3^. (5) A data-dependent program was used in analysis so that the two most abundant ions in each scan were selected and subjected to MS^2^ and MS^3^ analyses. (6) All data were acquired and processed using Shimadzu LCMS solution Version 3.36, Formula Predictor Version 1.01, and Accurate Mass Calculator software (Shimadzu Corporation, Guangzhou, China, LCMS solution, version 3). (7) Mass calibration was carried out using a trifluoroacetic acid sodium solution (2.5 mmol/L) from 50 to 1000 Da.

### 3.8. Spectroscopic Methods

UV spectra (200–400 nm) were recorded using an LCMS-IT-TOF instrument with a DAD detector. High-resolution mass spectrography data were obtained using an LCMS-IT-TOF instrument with a TOF mass analyzer or a Bruker Apex IV FT-MS (7.0T). One- and two-dimensional NMR spectra were obtained on a Bruker DRX 400 spectrometer with a 5 mm probe at room temperature. Metabolites were dissolved in 0.5 mL dimethyl sulfoxide-*d*_6_ (DMSO-*d*_6_) containing tetramethylsilane (TMS) as an internal standard. Chemical shifts (δ) were reported in ppm and coupling constants (*J*) were reported in Hertz.

### 3.9. Research on Network Pharmacology and Macromolecular Docking

Firstly, the Isometric SMILES numbers of the isolated metabolites were pasted onto the SwissTargetPrediction to obtain the corresponding targets for each compound. Then, the GeneCards database was searched for potential targets of cardiovascular diseases, and then Venny map of compounds and cardiovascular targets was made on the Venny2.1 website to obtain intersection targets, and the intersection targets were pasted on String database and the software Cytoscape 3.9.1 for PPI network mapping. The intersection targets with Betweenness Centrality values of 0 and those below 85 were rounded off, and the Betweenness Centrality values from high to low were divided into three parts. The innermost layer was the target with the largest Betweenness Centrality value, and the topological map of the intersection target of active ingredient and cardiovascular disease of Betweenness Centrality value was derived. Finally, based on the above research results, macromolecular docking verification was carried out, and compounds with higher selection values were selected as ligands and core targets as receptors for macromolecular docking [20,21,22,23]. In macromolecular docking, it is generally believed that a binding energy between ligand and protein < −4.25 kcal/mol indicates that there is a specific binding activity between them. A Binding energy < −5.0 kcal/mol indicates that they have good binding activity. A Binding energy < −7.0 kcal/mol indicates that the ligand has strong binding activity to the receptor [22,23].

## 4. Conclusions

In conclusion, this study focuses on the metabolism of formononetin and its glycosides (onion) in rats, both in vivo and in vitro. Firstly, 6 metabolites of formononetin were isolated from rat liver S9 after incubation in vitro, and 14 possible metabolites were identified through high-resolution mass spectrometry. Additionally, 21 possible metabolites were identified through high-resolution mass spectrometry in the collected rat urine samples after the in vivo administration of onion. The results indicate that the main forms of formononetin and its glycosides (onion) after metabolism are basically similar, except for the binding reactions. This suggests that the metabolism of formononectin primarily occurs in the livers of rats. The six metabolites obtained represent the main form of formononetin metabolism due to the higher content of similar isoflavone metabolites; it also indicates that they are the main active components responsible for producing pharmacological effects. Due to the fact that a prototype drug may produce a large number of metabolites after administration, the prototype or metabolites may play therapeutic roles, which require a specific network pharmacological analysis. Finally, the above six metabolites isolated from rat liver S9 incubated in vitro by formononetin were analyzed via network pharmacology and macromolecular docking technology, and 10 receptors related to CVD were found. Among them, HSP90AA and SRC showed significant impacts based on their binding energies. In conclusion, this study found that formononetin and its main metabolites, Sm10 and Sm12, may have roles in the treatment of CVD due to their good binding activities with the HSP90AA1 receptor, while Sm7 may have a role due to its good binding activity with the SRC receptor. In the future, research will involve animal and clinical experiments to further validate these findings.

## Figures and Tables

**Table 1:** The ^1^H and ^13^C-NMR spectra of metabolites of formononetin.

	Formononetin		Sm7		Sm5		Sm10		Sm9		Sm2	
	δ_C_	δ_H_ (*J* = Hz)	δ_C_	δ_H_ (*J* = Hz)	δ_C_	δ_H_ (*J* = Hz)	δ_C_	δ_H_ (*J* = Hz)	δ_C_	δ_H_ (*J* = Hz)	δ_C_	δ_H_ (*J* = Hz)
1												
2	153.5	8.32 s	153.3	8.30 s	153.4	8.37 s	153.1	8.27 s	153.4	8.31 s	152.7	8.22 s
3	123.6		123.0		123.0		122.8		123.1		123.2	
4	175.0		175.1		175.5		174.7		175.6		174.8	
5	127.7	7.94d (9)	127.7	7.94d (8)	116.1	7.45d (8)	108.5	7.37 s	116.1	7.44d (8)	108.6	7.36 s
6	115.6	6.91dd (2,8)	115.6	6.90dd (2,8)	114.7	6.94d (8)	145.1	9.77 s	114.6	6.92d (8)	145.1	9.48 s
7	163.0	10.78 s	162.9	10.76 s	150.5	10.31 s	151.3	10.41 s	150.4	10.28 s	151.3	10.38 s
8	102.6	6.85d (2)	102.5	6.85d (2)	133.4	9.42 s	103.2	6.89 s	133.3	9.41 s	103.2	6.87 s
9	157.9		157.6		147.2		152.7		147.2		152.8	
10	117.1		117.1		117.9		117.0		117.9		117.0	
1′	124.7		123.9		124.8		125.0		123.4		123.4	
2′	130.5	7.48d (8)	130.5	7.35d (8)	130.6	7.49d (8)	130.5	7.47d (8)	130.6	7.36d (8)	130.5	7.34d (8)
3′	114.1	6.96d (8)	115.4	6.78 (8)	114.0	6.96 (8)	114.0	6.95 (8)	115.4	6.78 (8)	115.3	6.77d (8)
4′	159.4		157.9	9.50 s	159.4		159.3		157.6	9.50 s	157.5	9.75 s
5′	114.1	6.96d (8)	115.4	6.78 (8)	114.0	6.96 (8)	114.0	6.95 (8)	115.4	6.78 (8)	115.3	6.77d (8)
6′	130.5	7.48d (8)	130.5	7.35d (8)	130.6	7.49d (8)	130.5	7.47d (8)	130.6	7.36d (8)	130.5	7.34d (8)
-OCH3	55.6	3.77 s			55.6	3.77 s	55.6	3.77 s				

**Table 2:** Proposed metabolites of formononetin in hepatic S9 incubated sample as indicated via LC/MS analysis.

Metabolites	t_R_ (min)	[M+H]^+^ (*m*/*z*)	[M−H]^−^ (*m*/*z*)	Formula	Error (ppm)	Main Fragment in MS^2^ or MS^3^	Metabolic Pathways
S0	31.205	269.0852	267.0669	C_16_H_12_O_4_	4.49	269, 237, 213, 163, 118, 107	Prototype (formononetin)
Sm1	18.568	447.1289	445.1134	C_22_H_22_O_10_	−0.45	447, 285(−162), 229, 152	hydroxylation, glycosylation
Sm2	19.663	271.0597	269.0452	C_15_H_10_O_5_	−3.32	271, 253, 225, 215, 197, 169, 153	hydroxylation, demethylation
Sm3	20.413		445.1115	C_22_H_22_O_10_	−4.49		hydroxylation, glycosylation
Sm4	20.897	431.1337		C_22_H_22_O_9_	−1.16	431, 269(−162), 237, 213, 181, 152, 136	glycosylation
Sm5	21.483	301.0699	299.0560	C_16_H_12_O_6_	1.34	301, 286, 269, 241, 229, 153	dihydroxylation
Sm6	22.458	417.1179	415.1011	C_21_H_20_O_9_	−4.34	417, 285(−132), 268, 152, 124	hydroxylation, glycosylation (pentose)
Sm7	22.847	255.0659	253.0493	C_15_H_10_O_4_	0.78	255, 227, 199, 137, 152, 109	demethylation
Sm8	23.227	417.1188	415.1025	C_21_H_20_O_9_	−0.96	417, 285(−132), 229, 152,	hydroxylation, glycosylation (pentose)
Sm9	23.425	271.0606		C_15_H_10_O_5_	−3.32	271, 253, 225, 215, 197, 169, 153	hydroxylation, demethylation
Sm10	24.080	285.0755	283.0599	C_16_H_12_O_5_	−2.81	285, 253, 225, 197, 141	hydroxylation
Sm11	25.132	417.1193	415.1055	C_21_H_20_O_9_	1.68	417, 285(−132), 253, 152, 123	hydroxylation, glycosylation (pentose)
Sm12	27.053	285.0811	283.0611	C_16_H_12_O_5_	1.77	285, 211, 183, 152	hydroxylation
Sm13	27.518	285.0756	283.0604	C_16_H_12_O_5_	−2.46	285, 270, 229, 211, 197, 183, 152	hydroxylation
Sm14	32.385	299.385		C_17_H_14_O_5_	0.33	299, 270, 254, 237, 213, 181	hydrolation, methylation

**Table 3:** Proposed metabolites of ononin in rat biological samples as indicated via LC/MS analysis.

Metabolites	t_R_ (min)	[M+H]^+^ (*m*/*z*)	[M−H]^−^ (*m*/*z*)	Formula	Error (ppm)	Main Fragment in MS^2^ or MS^3^	Metabolic Pathways
M1	33.707	431.0954	429.0815	C_21_H_18_O_10_	−1.63	429, 253(−176), 224, 208, 175, 135	demethylation, glucuronidation
M2	34.397		431.0982	C_21_H_20_O_10_	0.93	431, 255(−176), 175, 149	hydrogenation, demethylation, glucuronidation
M3	35.872		429.0833	C_21_H_18_O_10_	2.56	429, 253(−176), 224, 175	demethylation, glucuronidation
M4	36.372		431.0967	C_21_H_20_O_10_	−2.55		hydrogenation, demethylation, glucuronidation
M5	38.525		459.0938	C_22_H_20_O_11_	2.40	459, 283(−176), 268	hydroxylation, glucuronidation
M6	39.447		417.1188	C_21_H_22_O_9_	0.48		hydrogenation, carbonyl reduction, glucuronidation
M7	39.705		417.1181	C_21_H_22_O_9_	−1.20		hydrogenation, carbonyl reduction, glucuronidation
M8	42.007	461.1097	459.0923	C_22_H_20_O_11_	2.82	461, 285(−176), 270, 152, 123	hydroxylation, glucuronidation
M9	42.627	255.0668	253.0525	C_15_H_10_O_4_	4.31	255, 199, 152, 137	demethylation
M10	43.143	445.1127	443.0983	C_22_H_20_O_10_	−1.80	445, 269(−176), 237, 118	glucuronidation
M11	43.445	445.1114	443.0988	C_22_H_20_O_10_	−4.72	443, 267(−176), 152, 175	glucuronidation
M12	43.703	475.1224	473.1077	C_23_H_22_O_11_	−3.37		methylation, hydroxylation, glucuronidation
M13	44.168	447.1294	445.1158	C_22_H_22_O_10_	0.67	445, 269(−176), 254, 175, 135	hydrogenation, demethylation, glucuronidation
M14	44.988	285.0778	283.0611	C_16_H_12_O_5_	5.26	283, 268, 224, 131	hydroxylation
M15	45.652	461.1067	459.0924	C_22_H_20_O_11_	−0.65	461, 285(−176)	hydroxylation, glucuronidation
M16	48.427	285.0760	283.0617	C_16_H_12_O_5_	−1.05	283, 268, 224	hydroxylation
M17	48.935	285.0779		C_16_H_12_O_5_	5.61	285, 241	hydroxylation
M18	53.890	299.0905		C _17_H_14_O_5_	−4.68	299, 284, 243, 166, 137	hydroxylation, methylation
M19	67.932	335.0203	333.0083	C_15_H_10_O_7_S	1.19	333, 253(−80), 224, 135	demethylation, sulfonation
M20	76.412	335.0229	333.0092	C_15_H_10_O_7_S	4.20	333, 253(−80), 225, 211, 135	demethylation, sulfonation
M21	52.977	269.0847	267.0654	C_16_H_12_O_4_	−1.12		formononetin

**Table 4:** The top 10 cardiovascular-related disease targets.

No.	Names of the Receptors	Betweenness
1	HSP90AA1	4195.108
2	SRC	3675.1655
3	ESR1	2125.8835
4	HSPA8	1557.0778
5	APP	1246.3787
6	AHR	1209.1862
7	EGFR	1152.9003
8	PLG	1064.3462
9	CYP1B1	1034.5195
10	HDAC1	950.46075

**Table 5:** Macromolecular docking results of core components and corresponding core targets.

Six Isolated Metabolites	Binding Energy/kcal.mol^−1^	
	HSP90AA1	SRC
6,7,4′-trihydroxy-isoflavonoid (Sm2)	−4.04	−4.38
7,4′-dihydroxy-isoflavonoid (Sm7)	−4.76	−5.01
7,8,4′-trihydroxy-isoflavonoid (Sm9)	−4.18	−4.5
7,8-dihydroxy-4′-methoxy-isoflavonoid (Sm10)	−5.4	−4.06
6,7-dihydroxy-4′-methoxy-isoflavonoid (Sm12)	−5.16	−4.89
Formononetin (prototype)	−5.37	−4.37

## Data Availability

Available upon request and with regulations.

## Supplementary Materials

The following supporting information can be downloaded at: https://www.mdpi.com/article/10.3390/molecules28217451/s1, Figure S1. 1H and 13C NMR spectrum of Formononetin; Figure S2. 1H and 13C NMR spectrum of Ononin; Figure S3. 1H and 13C NMR spectrum of Sm2; Figure S4. 1H and 13C NMR spectrum of Sm7; Figure S5. 1H and 13C NMR spectrum of Sm9; Figure S6. 1H and 13C NMR spectrum of Sm10; Figure S7. 1H and 13C NMR spectrum of Sm12; Figure S8–S16 and Sm0-Sm14. MSn spectra of S9 with formononetin in PI mode; Figure S17–S23 and M1–M21. MSn spectra of ononin in urine sample in PI mode; Table S1. Proposed metabolites of formononetin in hepatic S9 incubated sample by LC/MS analysis; Table S2. Proposed metabolites of ononin in rat biological sample by LC/MS analysis.
