# Supplementary material for: Identification of the Metabolites of Both Formononetin in Rat Hepatic S9 and Ononin in Rat Urine Samples and Preliminary Network Pharmacology Evaluation of Their Main Metabolites
Source: Molecules. 2023 Nov 6;28(21):7451. doi: 10.3390/molecules28217451 (PMC10648658; doi:10.3390/molecules28217451)
Supplement: Supplementary file 1 [file molecules-28-07451-s001.zip › molecules-2629400-supplementary.pdf]

Supplemental data

Identification of the Metabolites of Both Formononetin in Rat Hepatic S9 and Ononin in Rat Urine Samples and Preliminary Network Pharmacology Evaluation of Their Main Metabolites

Yu-Zhu Yang [1,3,†], Tao Wang [2,†], Qi-Lei Chen [3], Hu-Biao Chen [3], Qian-Song He [4,*] and Ya-Zhou Zhang [1,3,*]

[1] College of Pharmacy, Guizhou University of Traditional Chinese Medicine, Guiyang 550025, China
[2] Departments of, Medicine and Biomedical Engineering, Faculty of Medicine and Health Sciences, McGill University, Montreal, QC H3G1Y6, Canada
[3] School of Chinese Medicine, Hong Kong Baptist University, Hong Kong SAR 999077, China
[4] First Clinical Medical College, Guizhou University of Traditional Chinese Medicine, Guiyang 550001, China
* Correspondence: heqiansong374@gzy.edu.cn (Q.-S.H.); zhangyazhou032@gzy.edu.cn (Y.-Z.Z.); Tel./Fax: +86-0851-8563-7296 (Q.-S.H.); Tel./Fax: +86-0851-8280-0211 (Y.-Z.Z.)
† These authors contributed equally to this work.

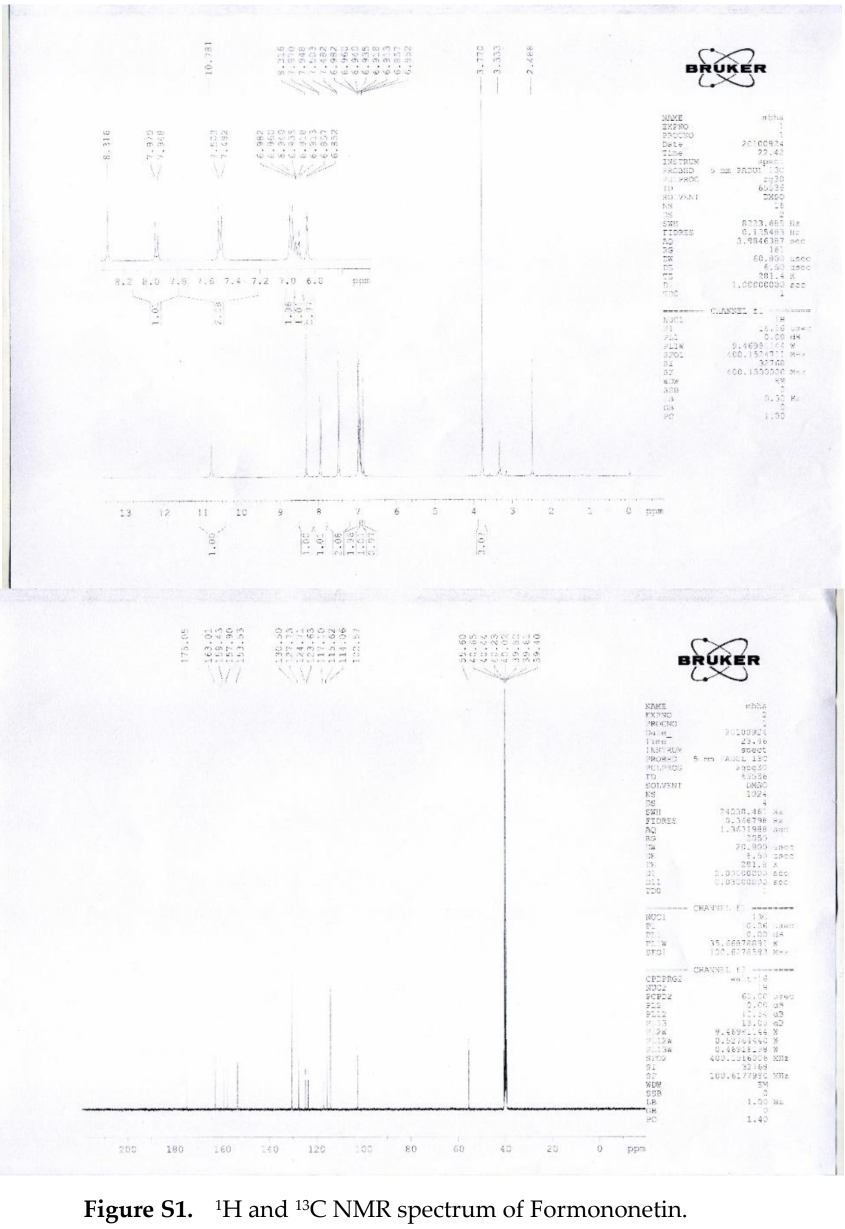

Figure S1. ^{1}H and ^{13}C NMR spectrum of Formononetin.

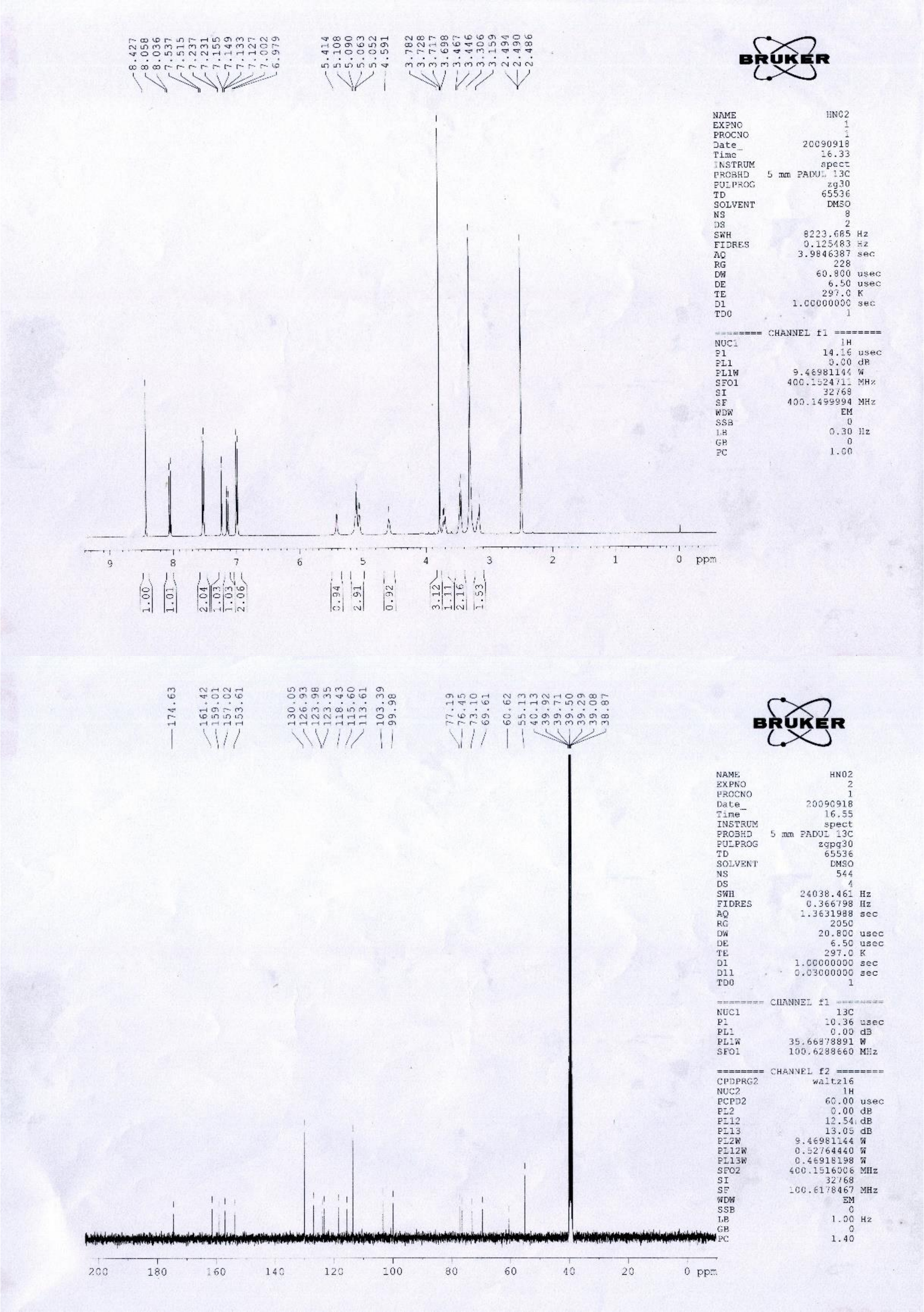

Figure S2. ^{1}H and ^{13}C NMR spectrum of Ononin.

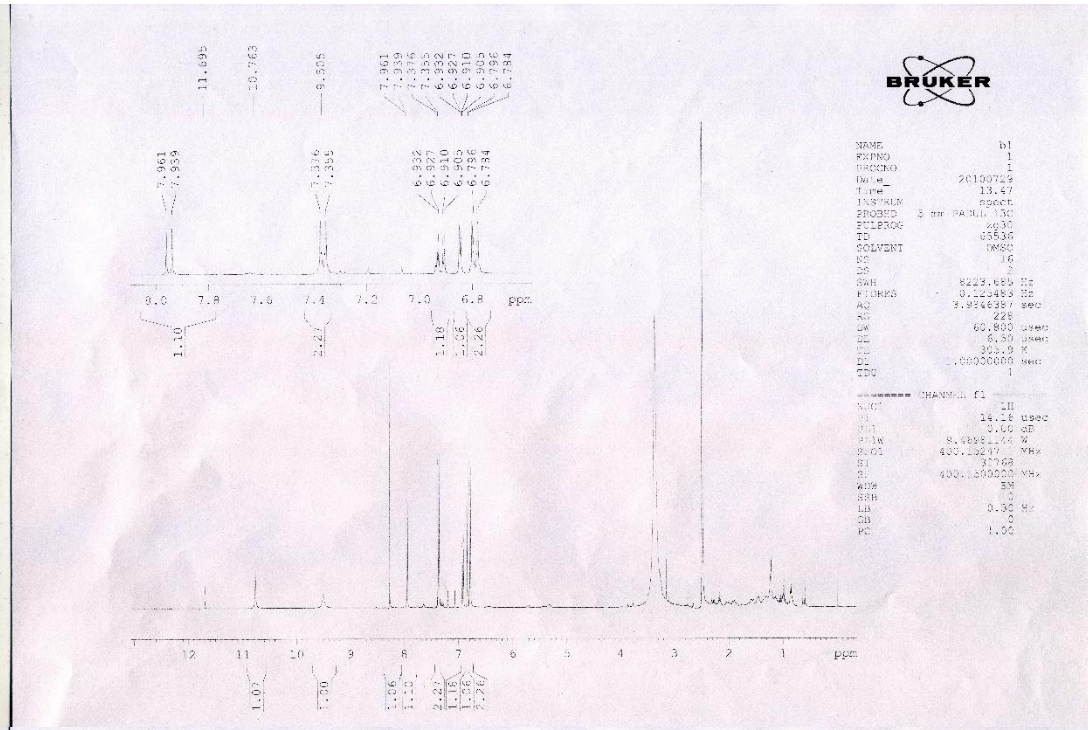

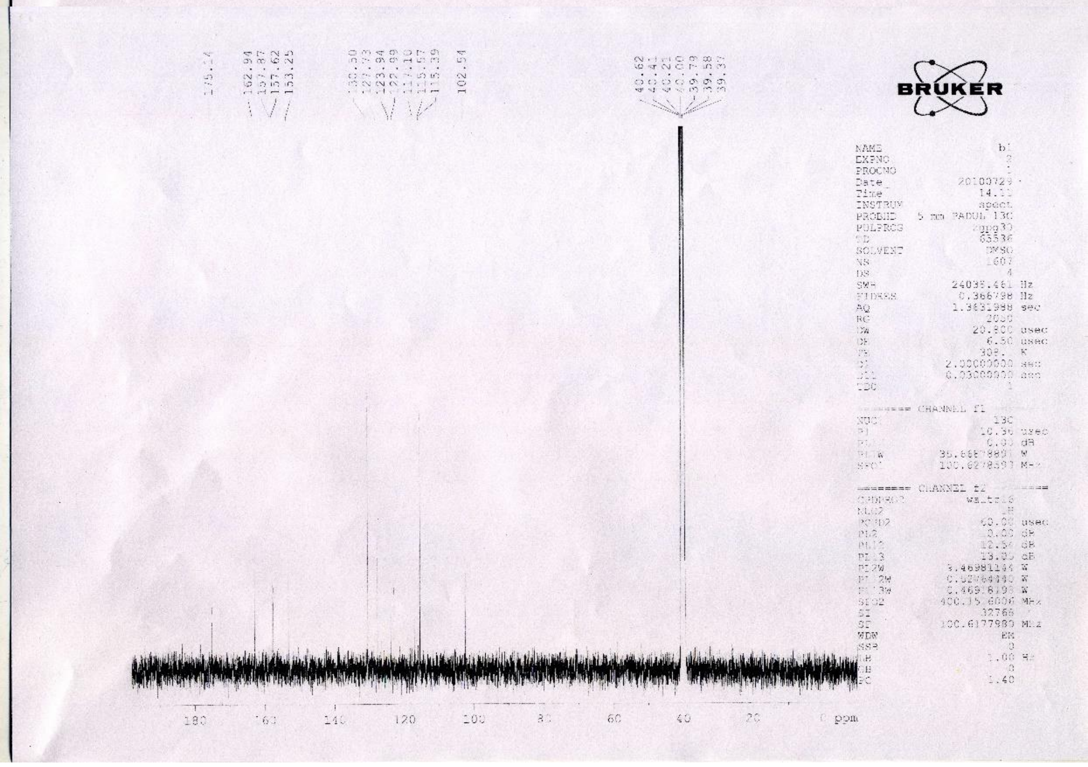

Figure S3. ^{1}H and ^{13}C NMR spectrum of Sm2.

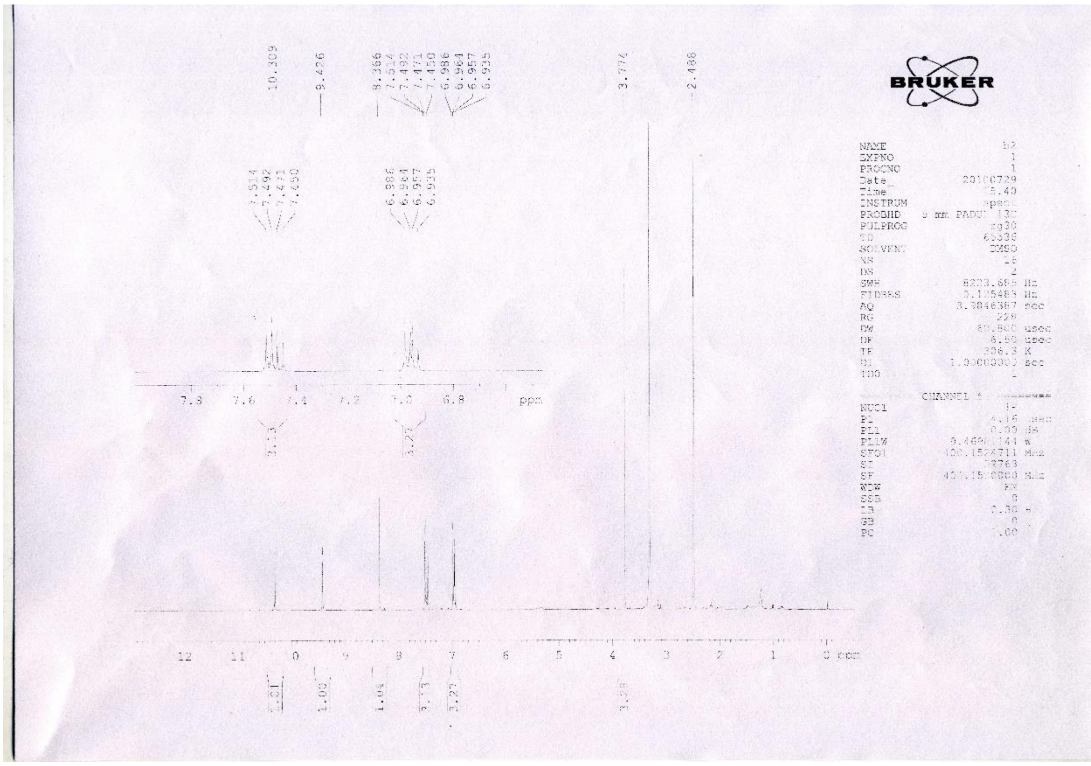

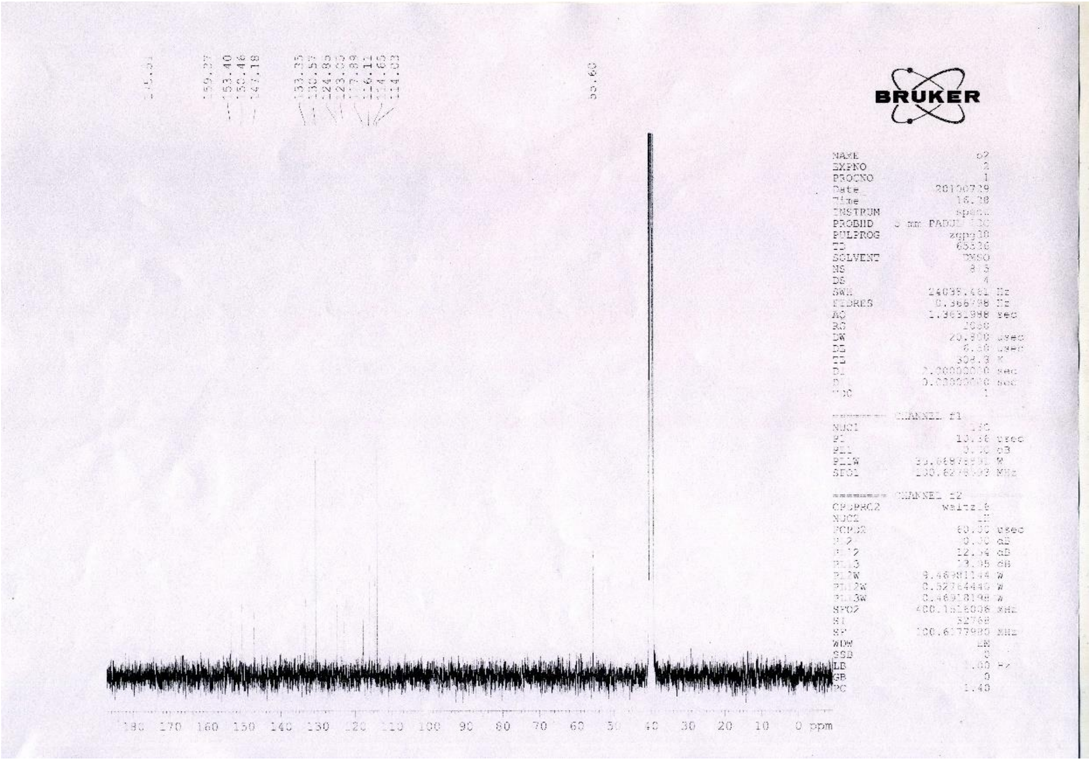

Figure S4. ^{1}H and ^{13}C NMR spectrum of Sm7.

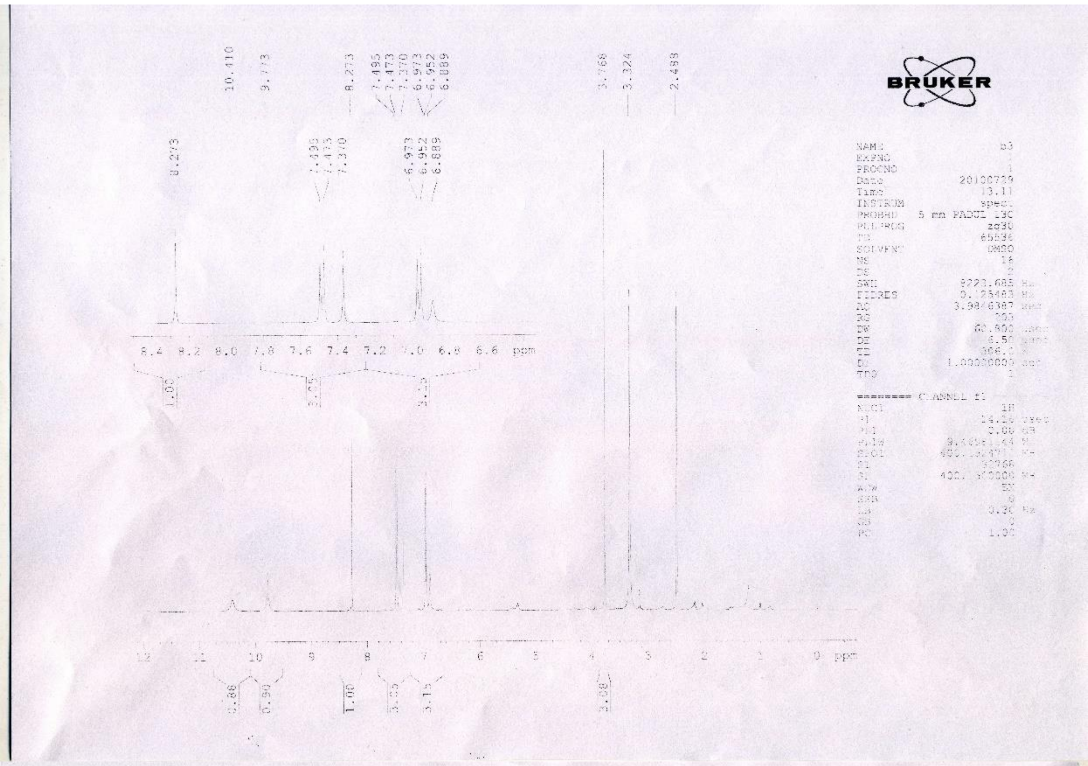

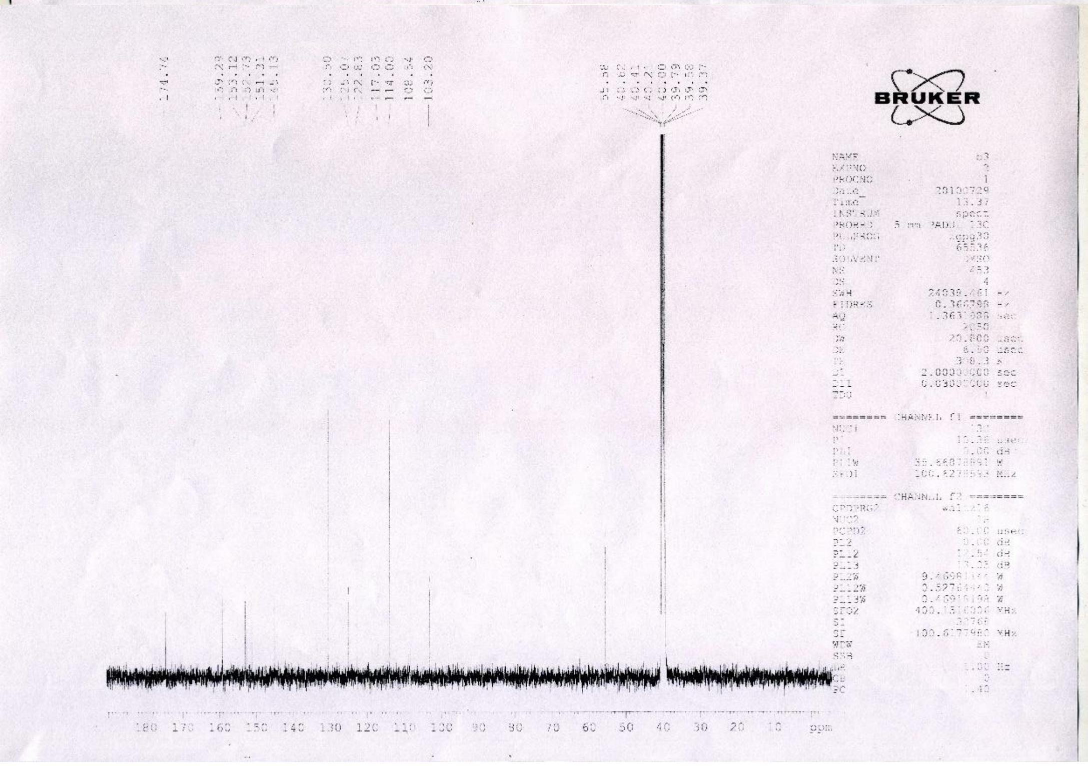

Figure S5. ^{1}H and ^{13}C NMR spectrum of Sm9.

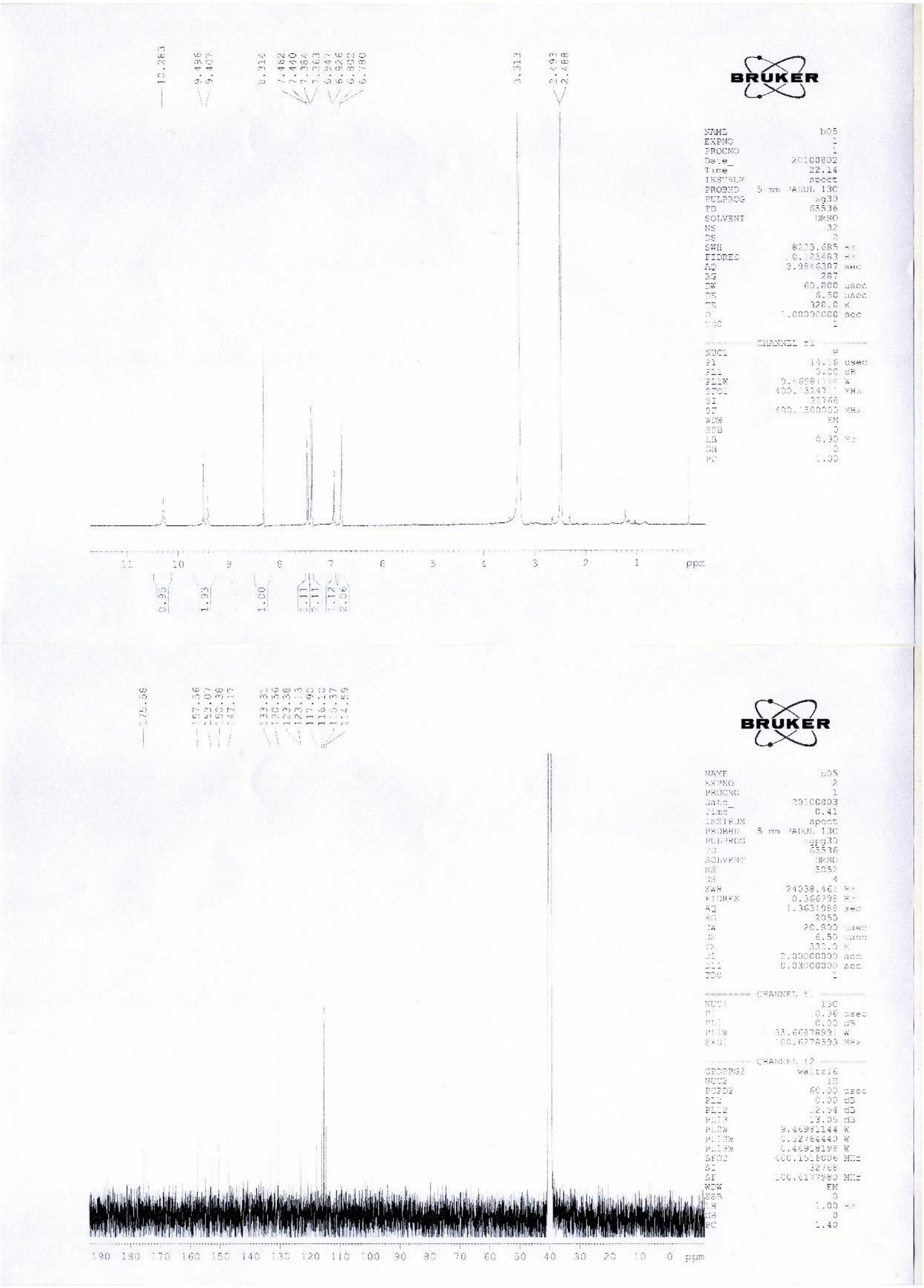

Figure S6. ^{1}H and ^{13}C NMR spectrum of Sm10.

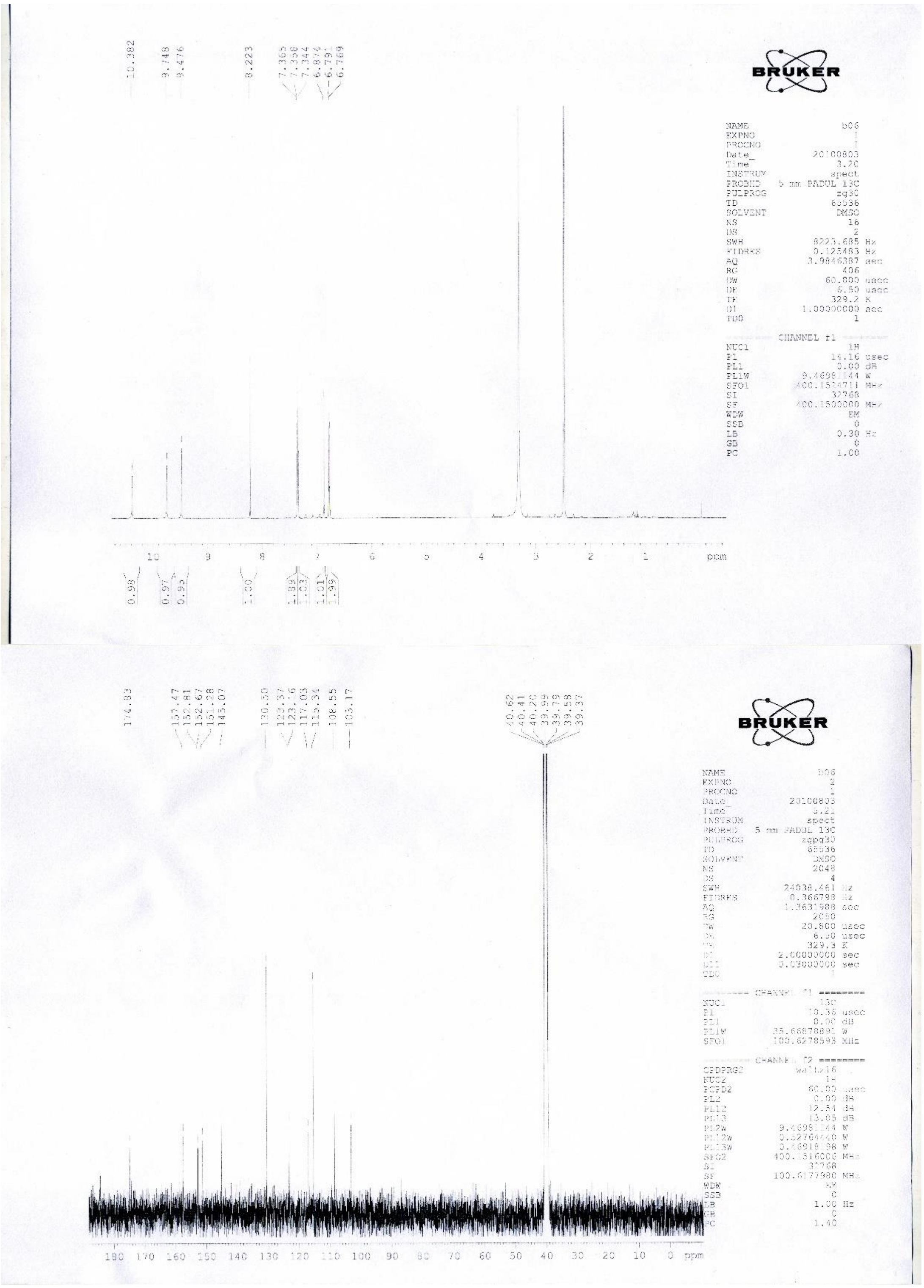

Figure S7. ^{1}H and ^{13}C NMR spectrum of Sm12.

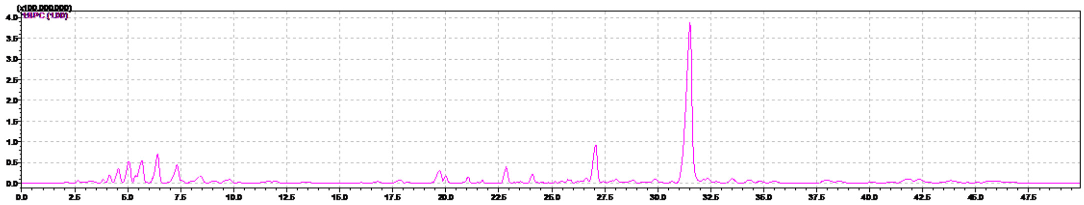

Figure S8. Formononetin with rats hepatic S9 samples by LC/MS analysis PI BPC.

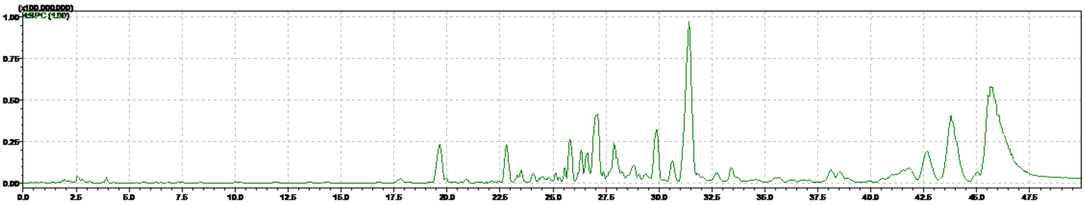

Figure S9. Formononetin with rats hepatic S9 samples by LC/MS analysis NI BPC.

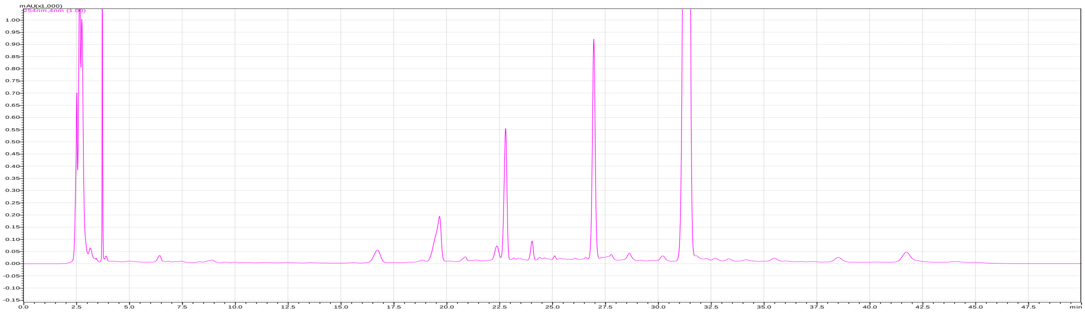

Figure S10. Formononetin with rats hepatic S9 samples UV(254nm).

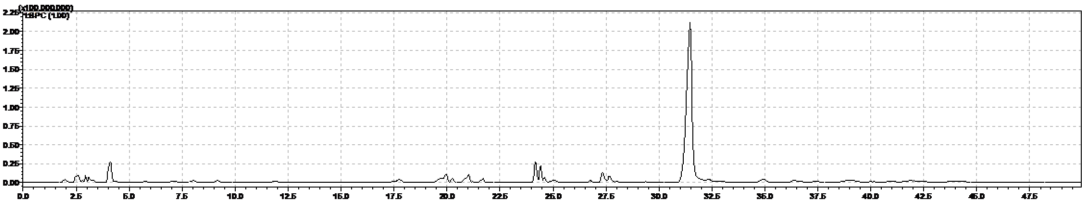

Figure S11. Formononetin without rats hepatic S9 samples PI BPC.

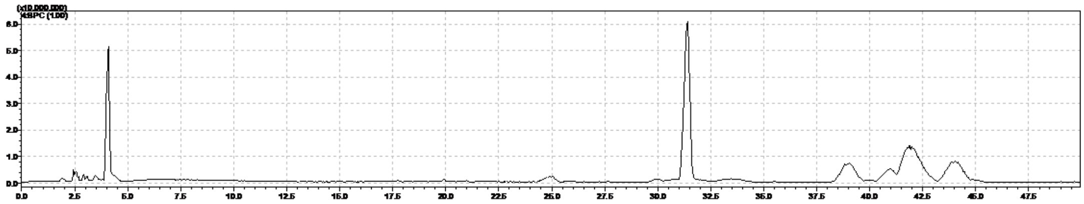

Figure S12. Formononetin without rats hepatic S9 samples NI BPC.

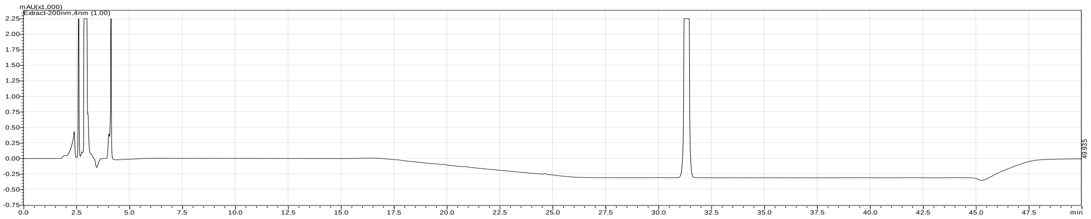

Figure S13. Formononetin without rats hepatic S9 samples UV(254nm).

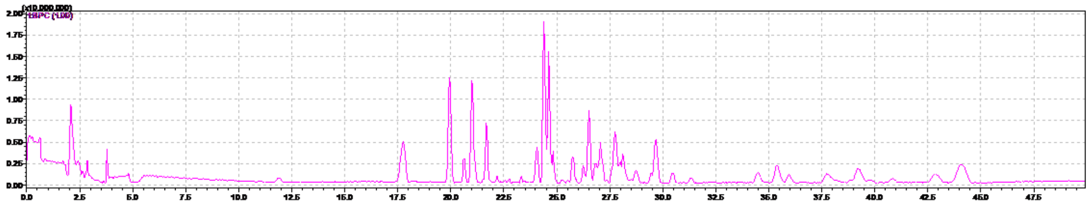

Figure S14. Hepatic S9 Sample PI BPC.

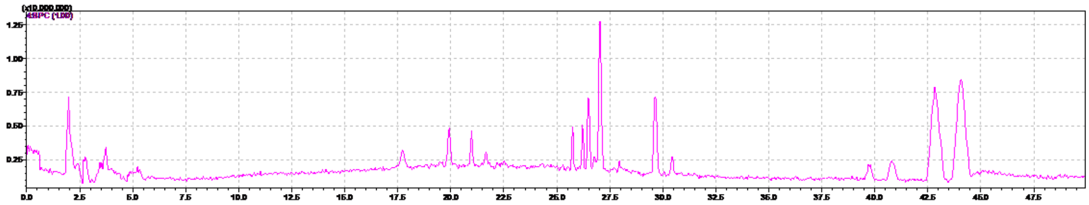

Figure S15. Hepatic S9 Sample NI BPC.

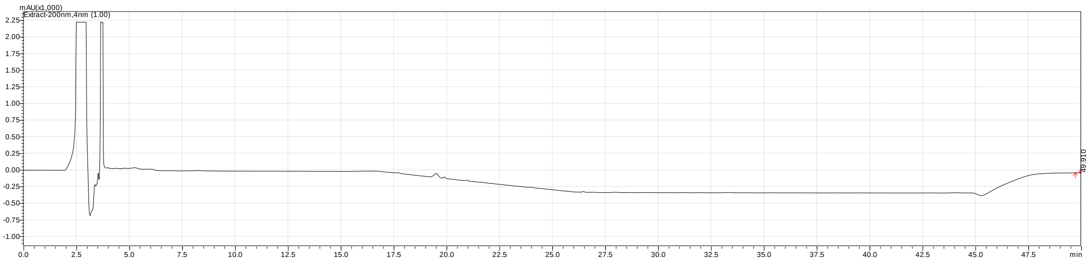

Figure S16. Hepatic S9 Sample UV(254nm).

Sm0 Formononetin

Event#: 1 MS(E+) Ret. Time : 31.205 Scan# : 3619

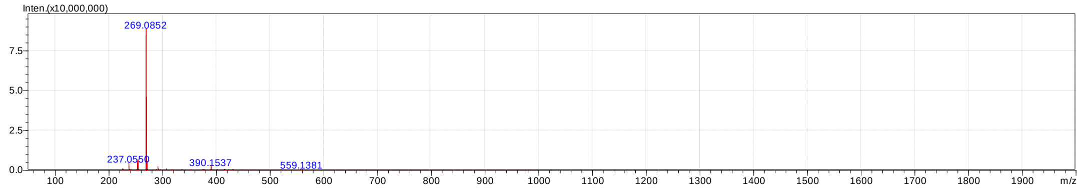

Event#: 4 MS(E-) Ret. Time : 31.205 Scan# : 3622

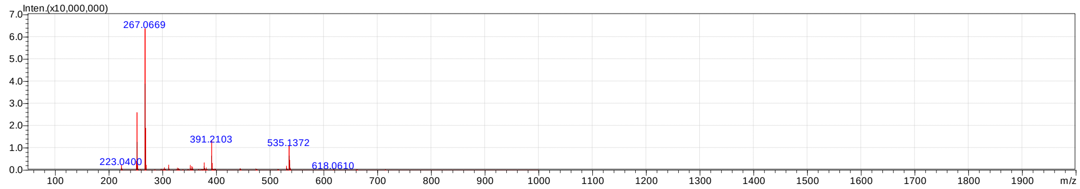

Rank	Score	Formula (M)	Ion	Meas. m/z	Pred. m/z	Diff (mDa)	Diff (ppm)	Iso Score	DBE
1	55.01	C16 H12 O4	[M-H]-	267.0669	267.0657	1.2	4.49	60.27	11.0

Sm1(metabolites)

Event#: 1 MS(E+) Ret. Time : 18.568 Scan# : 2153

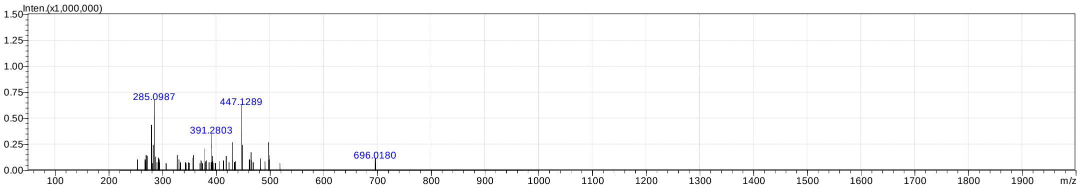

Event#: 4 MS(E-) Ret. Time : 18.568 Scan# : 2156

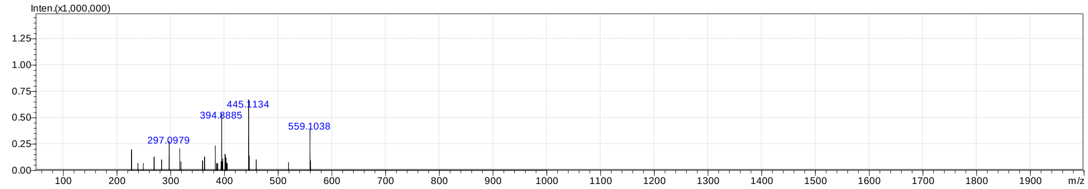

Rank	Score	Formula	(M)Ion	Meas. m/z	Pred. m/z	Diff (mDa)	Diff (ppm)	Iso Score	DBE
1	57.13	C22 H22 O10	[M+H]+	447.1289	447.1291	-0.2	-0.45	57.13	12.0

Sm2

Event#: 1 MS(E+) Ret. Time : 19.663 Scan# : 2280

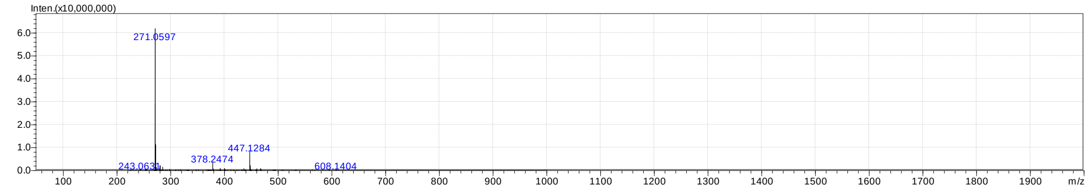

Event#: 4 MS(E-) Ret. Time : 19.663 Scan# : 2283

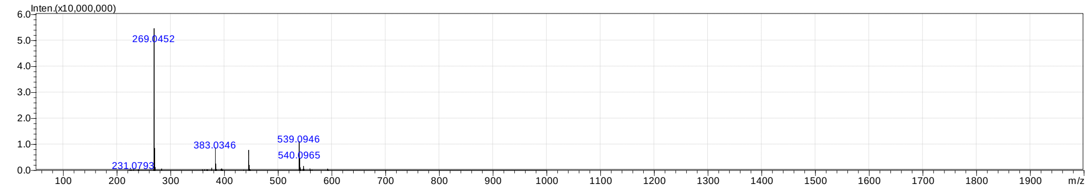

Rank	Score	Formula	(M)Ion	Meas. m/z	Pred. m/z	Diff (mDa)	Diff (ppm)	Iso Score	DBE
1	59.61	C15 H10 O5	[M+H]+	271.0597	271.0606	-0.9	-3.32	63.28	11.0

Sm3

Event#: 4 MS(E-) Ret. Time : 20.413 Scan# : 2370

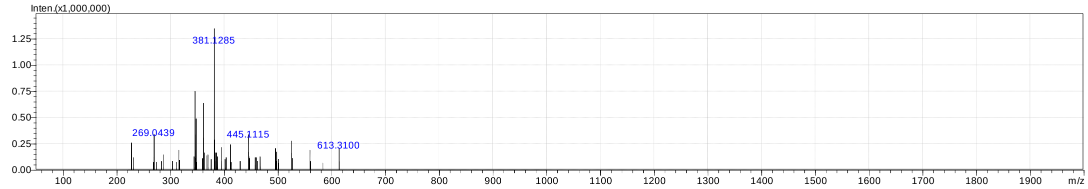

Rank	Score	Formula	(M)Ion	Meas. m/z	Pred. m/z	Diff (mDa)	Diff (ppm)	Iso Score	DBE
1	24.39	C26 H22 O5 S	[M-H]-	445.1115	445.1110	0.5	1.12	24.46	16.0
2	13.61	C22 H22 O10	[M-H]-	445.1115	445.1135	-2.0	-4.49	14.92	12.0

Sm4

Event#: 1 MS(E+) Ret. Time : 20.897 Scan# : 2423

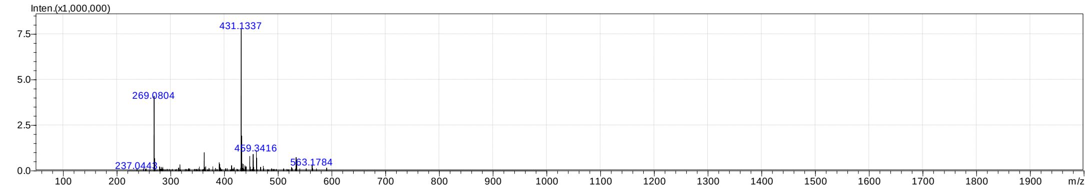

Event#: 4 MS(E-) Ret. Time : 20.897 Scan# : 2426

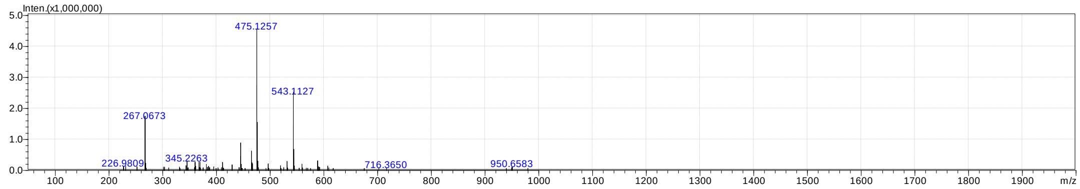

Rank	Score	Formula (M)Ion	Meas. m/z	Pred. m/z	Diff (mDa)	Diff (ppm)	Iso Score	DBE
1	96.59	C22 H22 O9 [M+H]+	431.1337	431.1342	-0.5	-1.16	96.98	12.0

Sm5

Event#: 1 MS(E+) Ret. Time : 21.483 Scan# : 2491

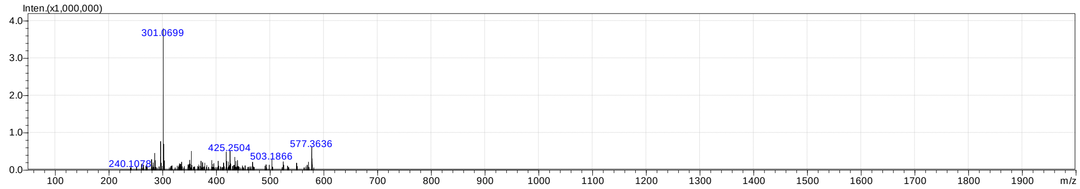

Event#: 4 MS(E-) Ret. Time : 21.483 Scan# : 2494

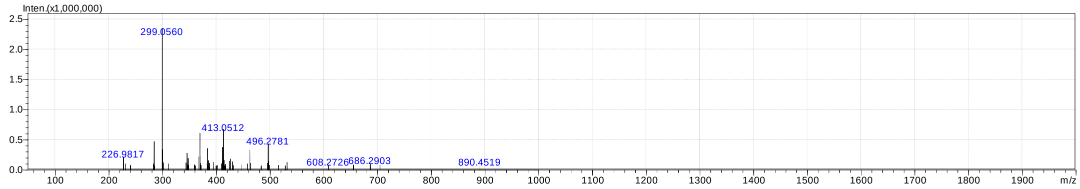

Rank	Score	Formula (M)Ion	Meas. m/z	Pred. m/z	Diff (mDa)	Diff (ppm)	Iso Score	DBE
1	90.94	C16 H12 O6 [M-H]-	299.0560	299.0556	0.4	1.34	91.72	11.0

Sm6

Event#: 1 MS(E+) Ret. Time : 22.458 Scan# : 2604

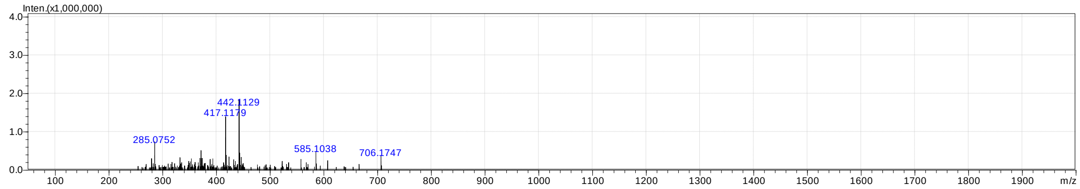

Event#: 4 MS(E-) Ret. Time : 22.458 Scan# : 2607

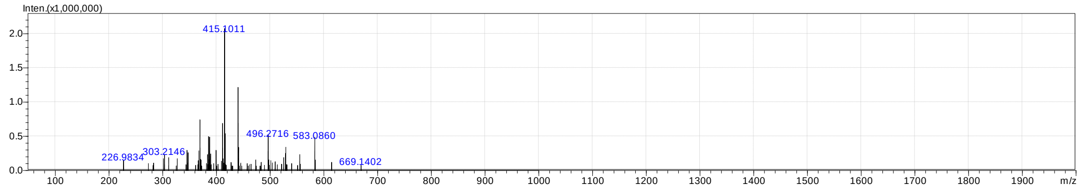

Rank	Score	Formula (M)Ion	Meas. m/z	Pred. m/z	Diff (mDa)	Diff (ppm)	Iso Score	DBE
1	71.25	C21 H20 O9 [M-H]-	415.1011	415.1029	-1.8	-4.34	77.75	12.0

Sm7

Event#: 1 MS(E+) Ret. Time : 22.847 Scan# : 2649

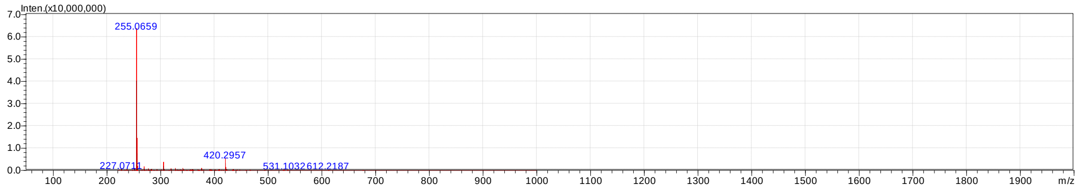

Event#: 4 MS(E-) Ret. Time : 22.847 Scan# : 2652

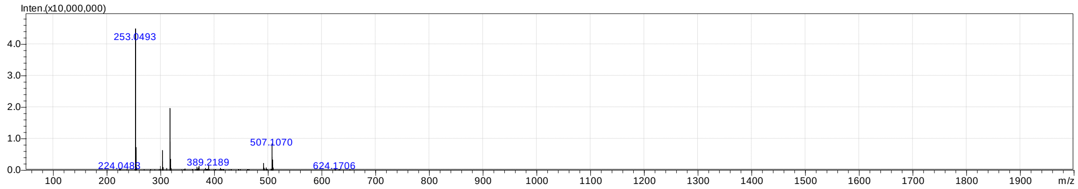

Rank	Score	Formula (M)Ion		Meas. m/z	Pred. m/z	Diff (mDa)	Diff (ppm)	Iso Score	DBE
1	89.33	C15 H10 O4	[M+H]+	255.0659	255.0657	0.2	0.78	89.33	11.0

Sm8

Event#: 1 MS(E+) Ret. Time : 23.227 Scan# : 2693

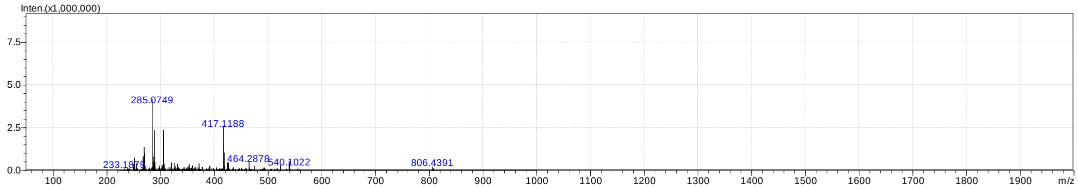

Event#: 4 MS(E-) Ret. Time : 23.227 Scan# : 2696

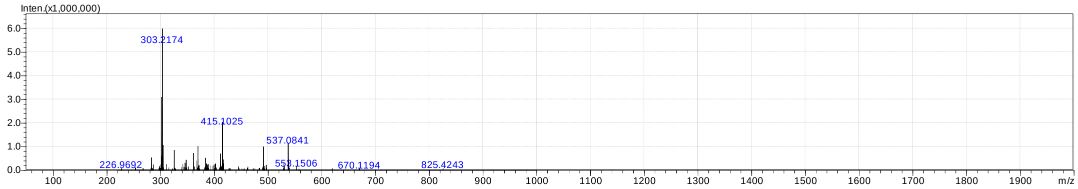

Rank	Score	Formula (M)Ion		Meas. m/z	Pred. m/z	Diff (mDa)	Diff (ppm)	Iso Score	DBE
1	65.43	C21 H20 O9	[M-H]-	415.1025	415.1029	-0.4	-0.96	65.43	12.0

Sm9

Event#: 1 MS(E+) Ret. Time : 23.425 Scan# : 2716

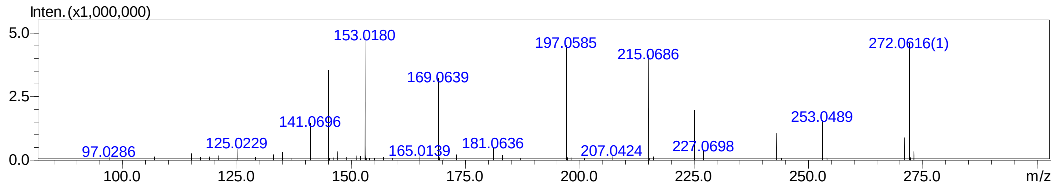

Rank	Score	Formula (M)Ion		Meas. m/z	Pred. m/z	Diff (mDa)	Diff (ppm)	Iso Score	DBE
1	67.08	C15 H10 O5	[M+H]+	271.0632	271.0645	-1.7	-3.32	72.13	12.0

Sm10

Event#: 1 MS(E+) Ret. Time : 24.080 Scan# : 2792

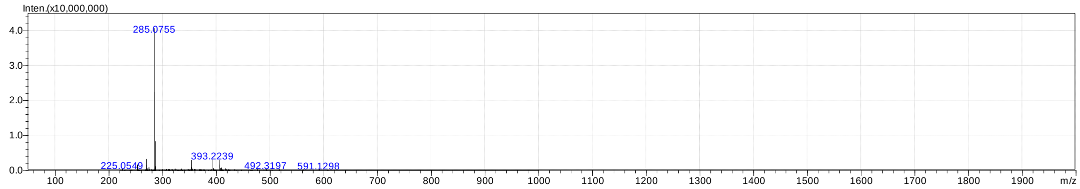

Event#: 4 MS(E-) Ret. Time : 24.080 Scan# : 2795

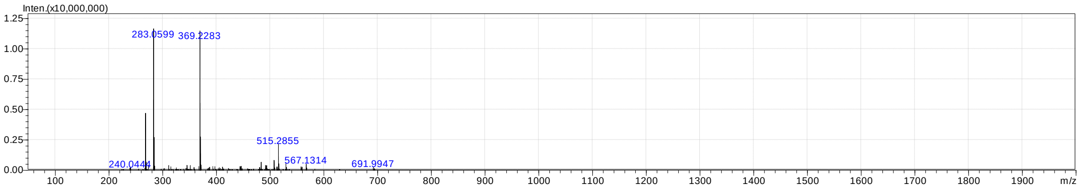

Rank	Score	Formula	(M)Ion	Meas. m/z	Pred. m/z	Diff (mDa)	Diff (ppm)	Iso Score	DBE
1	87.05	C16 H12 O5	[M+H]+	285.0755	285.0763	-0.8	-2.81	91.18	11.0

Sm11

Event#: 1 MS(E+) Ret. Time : 25.132 Scan# : 2914

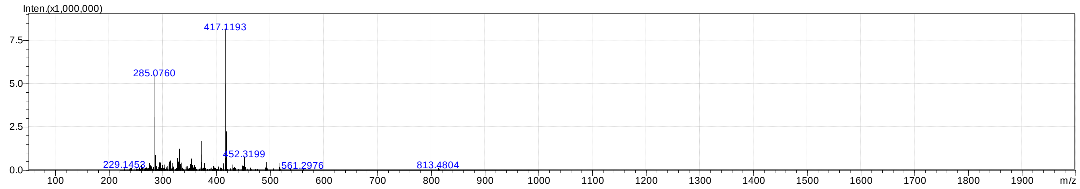

Event#: 4 MS(E-) Ret. Time : 25.132 Scan# : 2917

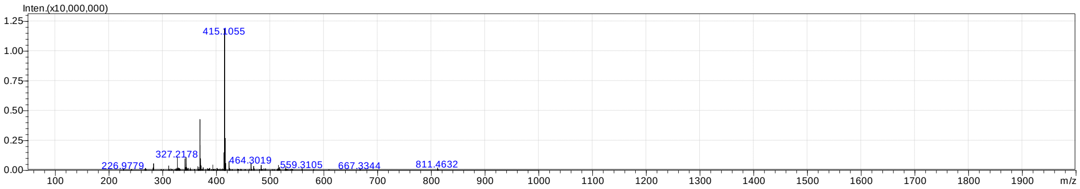

Rank	Score	Formula	(M)Ion	Meas. m/z	Pred. m/z	Diff (mDa)	Diff (ppm)	Iso Score	DBE
1	89.02	C21 H20 O9	[M+H]+	417.1193	417.1186	0.7	1.68	90.56	12.0

Sm12

Event#: 1 MS(E+) Ret. Time : 27.053 Scan# : 3137

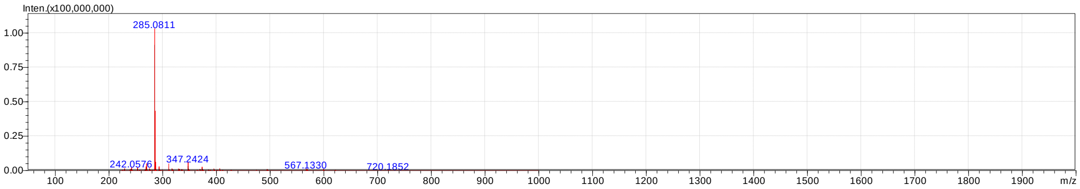

Event#: 4 MS(E-) Ret. Time : 27.053 Scan# : 3140

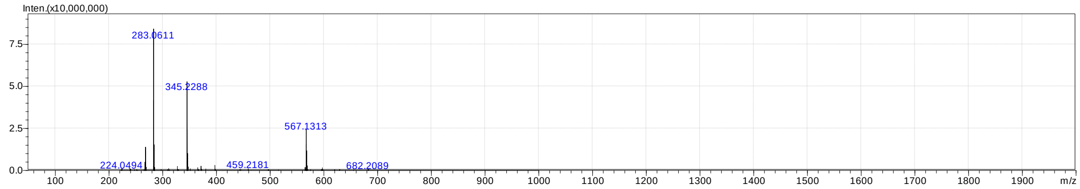

Rank	Score	Formula	(M)Ion	Meas. m/z	Pred. m/z	Diff (mDa)	Diff (ppm)	Iso Score	DBE

1	69.93	C16 H12 O5 [M-H]-	283.0611	283.0606	0.5	1.77	71.31	11.0

Sm13

Event#: 1 MS(E+) Ret. Time : 27.518 Scan# : 3191

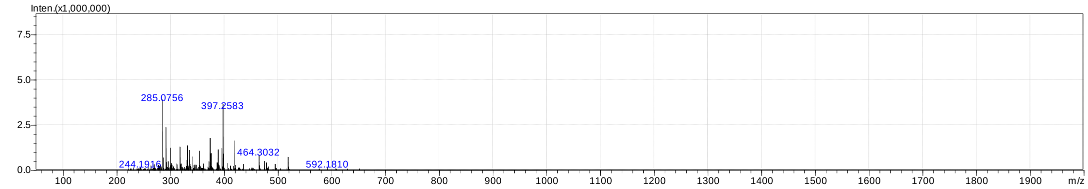

Event#: 4 MS(E-) Ret. Time : 27.518 Scan# : 3194

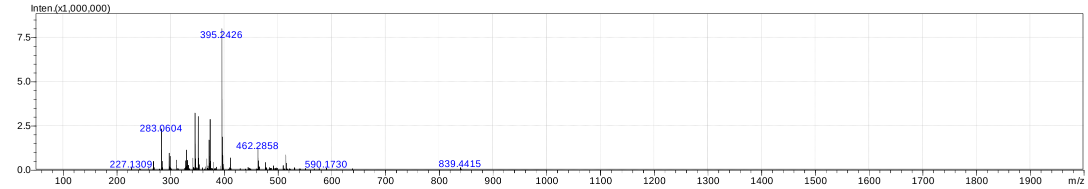

Rank	Score	Formula (M)Ion	Meas. m/z	Pred. m/z	Diff (mDa)	Diff (ppm)	Iso Score	DBE
1	89.33	C16 H12 O5 [M+H]+	285.0756	285.0763	-0.7	-2.46	92.71	11.0

Sm14

Event#: 1 MS(E+) Ret. Time : 32.385 Scan# : 3756

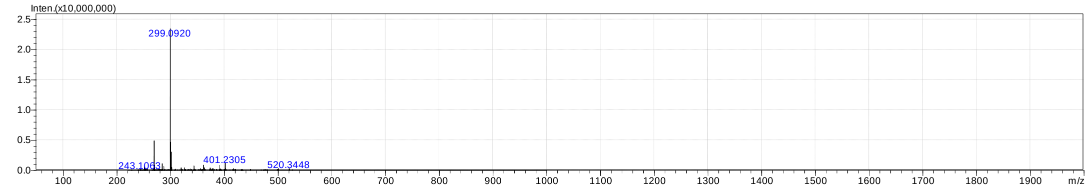

Rank	Score	Formula (M)Ion	Meas. m/z	Pred. m/z	Diff (mDa)	Diff (ppm)	Iso Score	DBE
1	45.63	C17 H14 O5 [M+H]+	299.0920	299.0919	0.1	0.33	45.63	11.0

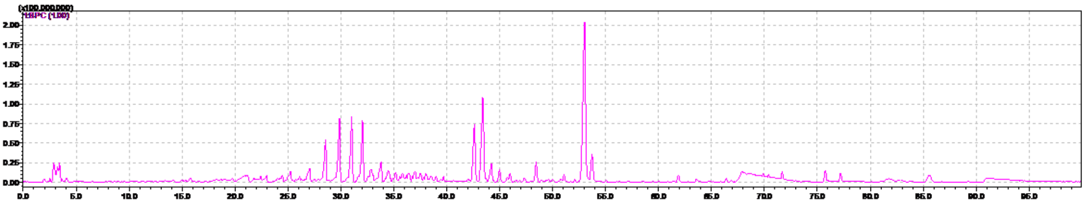

Figure S17. Onion in rat biological urine sample by LC/MS analysis PI BPC.

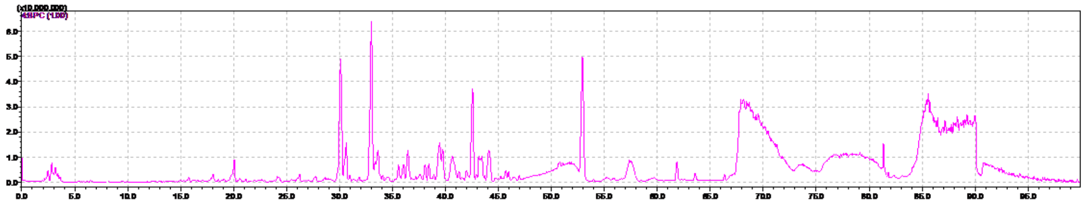

Figure S18. onion in rat biological urine sample by LC/MS analysis NI BPC.

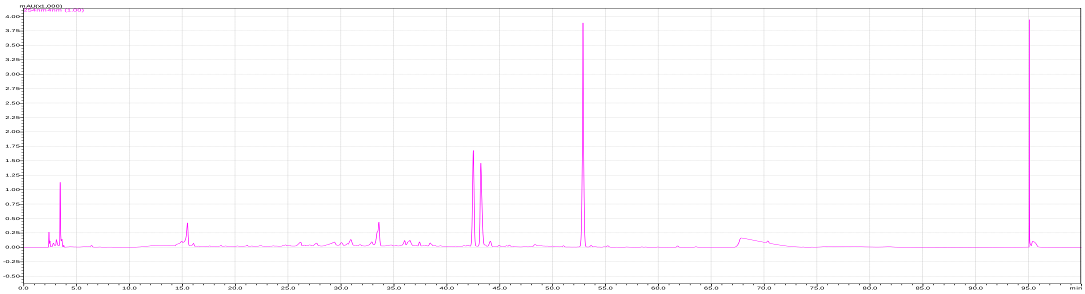

Figure S19. Onion in rat biological urine sample by LC/MS analysis UV(254nm).

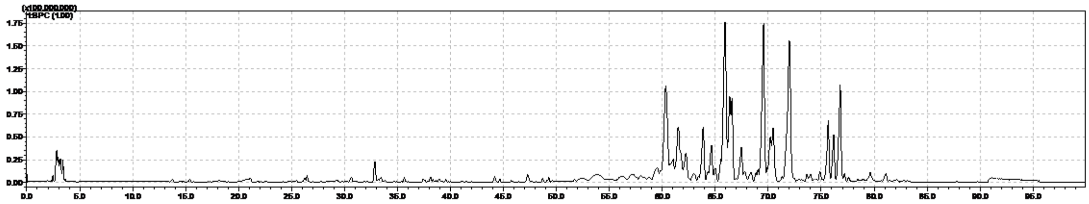

Figure S20. Rat biological blank urine sample by LC/MS analysis PI BPC.

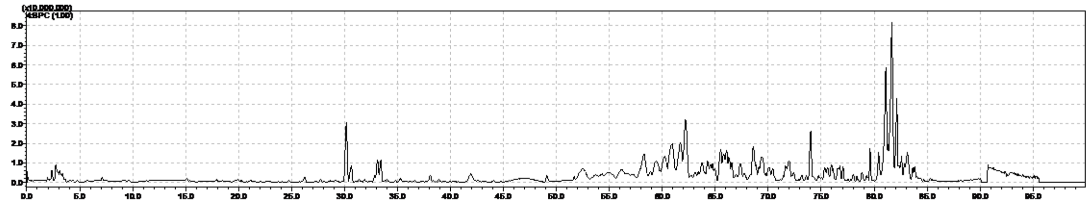

Figure S21. Rat biological blank urine sample by LC/MS analysis NI BPC.

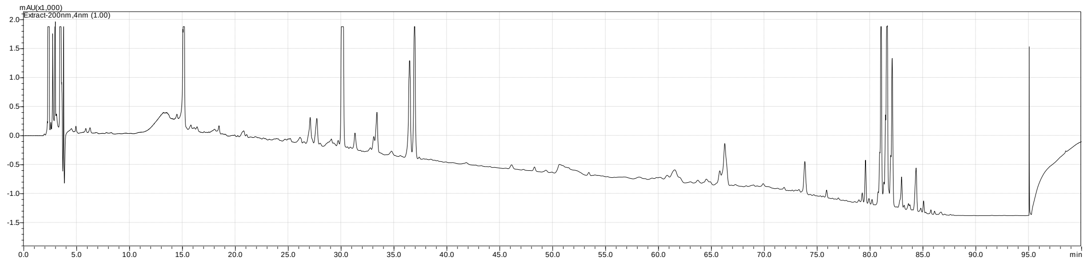

Figure S22. Rat biological blank urine sample by LC/MS analysis UV(254nm).

Onion

Event#: 1 MS(E+)   Ret. Time : 52.977   Scan# : 6144

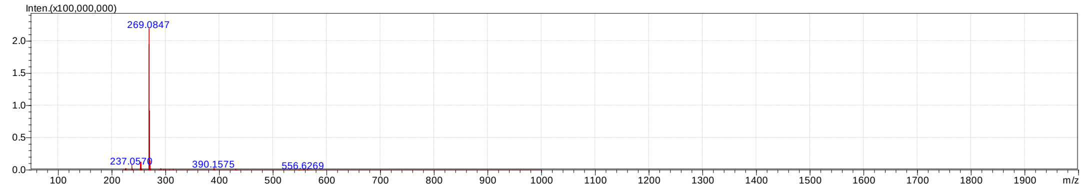

Event#: 4 MS(E-)   Ret. Time : 52.977   Scan# : 6147

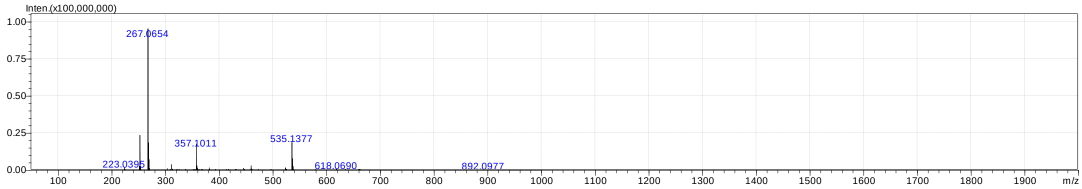

Rank	Score	Formula (M)Ion	Meas. m/z	Pred. m/z	Diff (mDa)	Diff (ppm)	Iso Score	DBE
1	57.38	C16 H12 O4 [M-H]-	267.0654	267.0657	-0.3	-1.12	57.55	11.0

M1(metabolite)

Event#: 1 MS(E+) Ret. Time : 33.707 Scan# : 3908

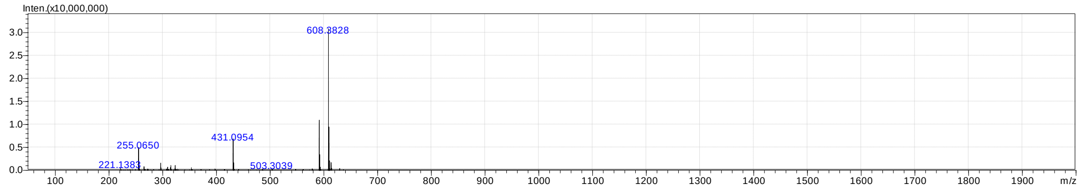

Event#: 4 MS(E-) Ret. Time : 33.707 Scan# : 3911

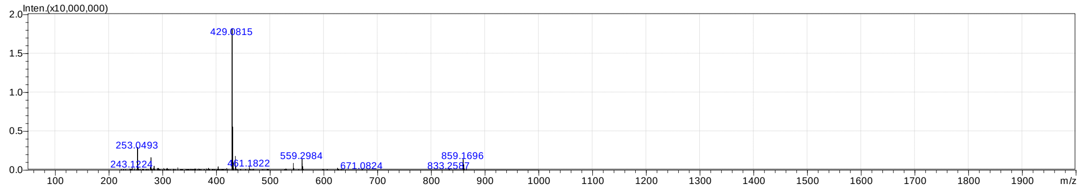

Rank	Score	Formula (M)Ion	Meas. m/z	Pred. m/z	Diff (mDa)	Diff (ppm)	Iso Score	DBE
1	79.79	C21 H18 O10[M-H]-	429.0815	429.0822	-0.7	-1.63	81.06	13.0

M2

Event#: 4 MS(E-) Ret. Time : 34.397 Scan# : 3991

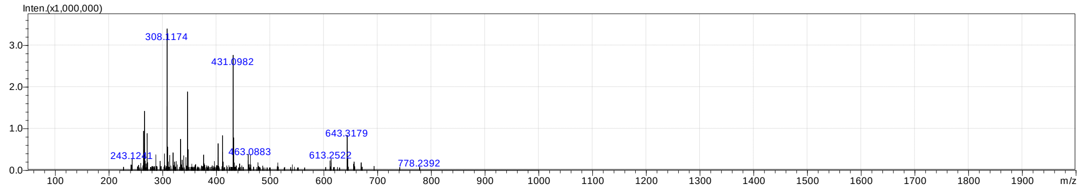

Rank	Score	Formula (M)Ion	Meas. m/z	Pred. m/z	Diff (mDa)	Diff (ppm)	Iso Score	DBE
1	45.52	C21 H20 O10 [M-H]-	431.0982	431.0978	0.4	0.93	45.52	12.0

M3

Event#: 4 MS(E-) Ret. Time : 35.872 Scan# : 4162

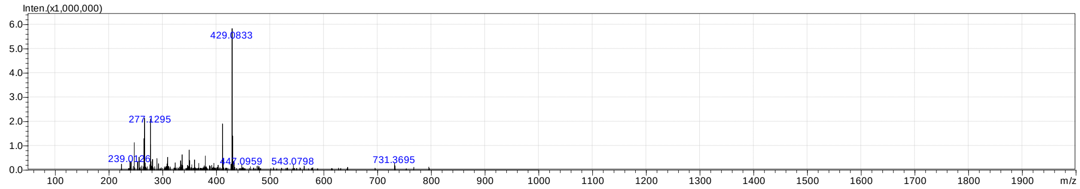

Rank	Score	Formula (M)Ion	Meas. m/z	Pred. m/z	Diff (mDa)	Diff (ppm)	Iso Score	DBE
1	75.60	C21 H18 O10 [M-H]-	429.0833	429.0822	1.1	2.56	78.67	13.0

M4

Event#: 4 MS(E-) Ret. Time : 36.372 Scan# : 4220

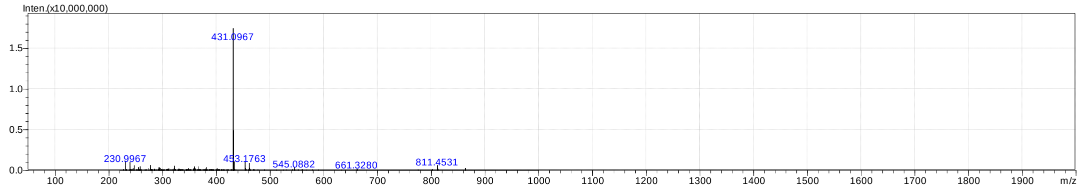

Rank	Score	Formula (M)Ion	Meas. m/z	Pred. m/z	Diff (mDa)	Diff (ppm)	Iso Score	DBE
1	74.11	C21 H20 O10 [M-H]-	431.0967	431.0978	-1.1	-2.55	77.10	12.0

M5

Event#: 4 MS(E-) Ret. Time : 38.525 Scan# : 4470

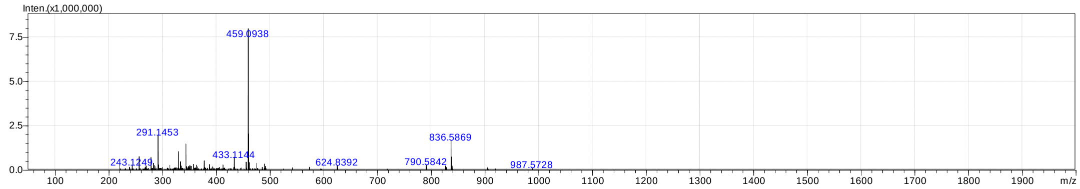

Rank	Score	Formula (M)Ion	Meas. m/z	Pred. m/z	Diff (mDa)	Diff (ppm)	Iso Score	DBE
1	90.87	C22 H20 O11 [M-H]-	459.0938	459.0927	1.1	2.40	94.17	13.0

M6

Event#: 4 MS(E-) Ret. Time : 39.447 Scan# : 4577

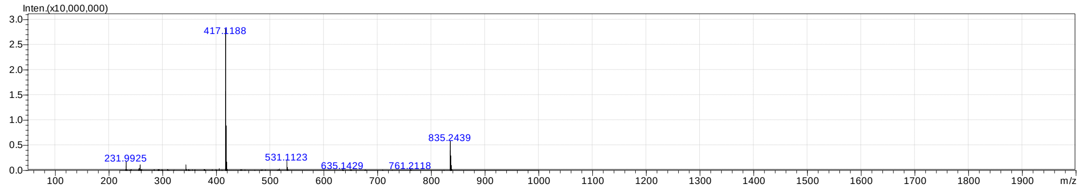

Rank	Score	Formula (M)Ion	Meas. m/z	Pred. m/z	Diff (mDa)	Diff (ppm)	Iso Score	DBE
1	85.77	C21 H22 O9 [M-H]-	417.1188	417.1186	0.2	0.48	85.77	11.0

M7

Event#: 4 MS(E-) Ret. Time : 39.705 Scan# : 4607

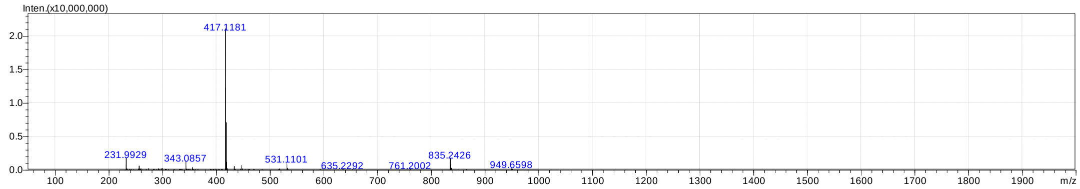

Rank	Score	Formula (M)Ion	Meas. m/z	Pred. m/z	Diff (mDa)	Diff (ppm)	Iso Score	DBE
1	84.43	C21 H22 O9 [M-H]-	417.1181	417.1186	-0.5	-1.20	84.86	11.0

M8

Event#: 1 MS(E+) Ret. Time : 42.007 Scan# : 4871

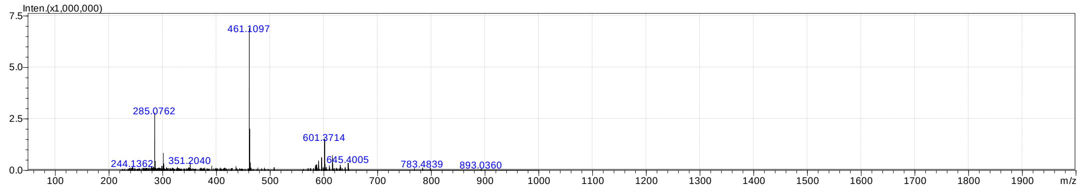

Event#: 4 MS(E-) Ret. Time : 42.007 Scan# : 4874

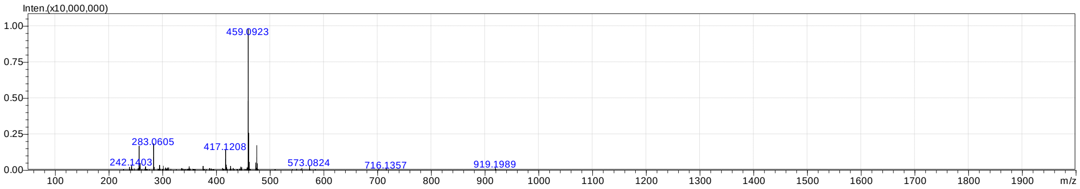

Rank	Score	Formula	(M)Ion	Meas. m/z	Pred. m/z	Diff (mDa)	Diff (ppm)	Iso Score	DBE
1	81.32	C22 H20 O11	[M+H]+	461.1097	461.1084	1.3	2.82	85.20	13.0

M9

Event#: 1 MS(E+) Ret. Time : 42.627 Scan# : 4943

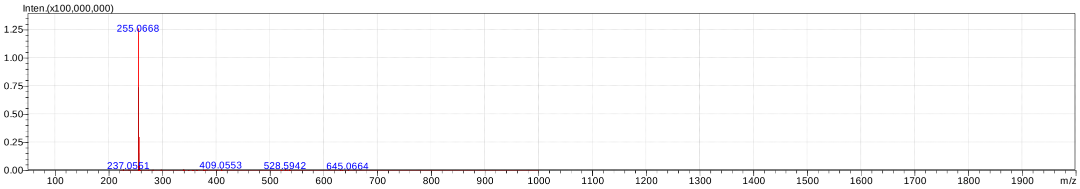

Event#: 4 MS(E-) Ret. Time : 42.627 Scan# : 4946

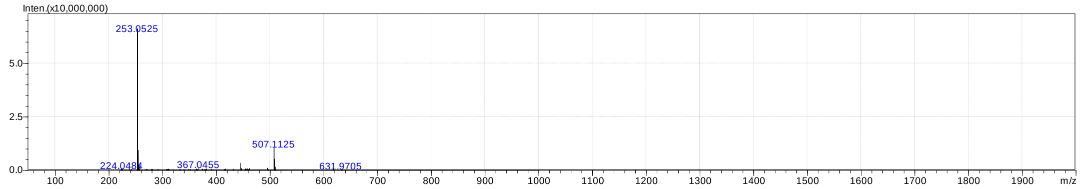

Rank	Score	Formula	(M)Ion	Meas. m/z	Pred. m/z	Diff (mDa)	Diff (ppm)	Iso Score	DBE
1	69.44	C15 H10 O4	[M+H]+	255.0668	255.0657	1.1	4.31	75.71	11.0

M10

Event#: 1 MS(E+) Ret. Time : 43.143 Scan# : 5003

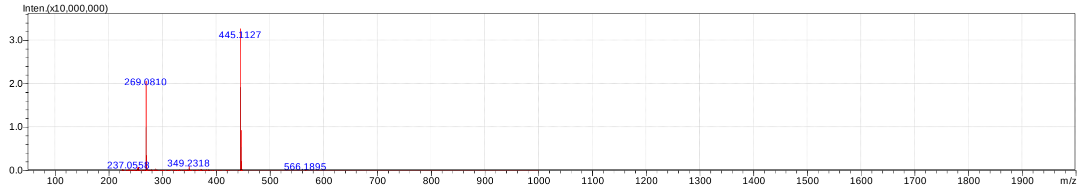

Event#: 4 MS(E-) Ret. Time : 43.143 Scan# : 5005

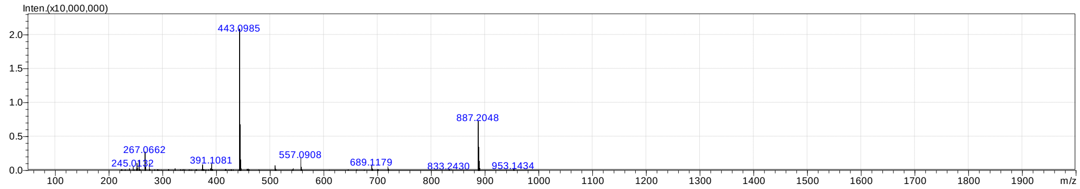

Rank	Score	Formula	(M)Ion	Meas. m/z	Pred. m/z	Diff (mDa)	Diff (ppm)	Iso Score	DBE

1	75.65	C22 H20 O10	[M+H]+	445.1127	445.1135	-0.8	-1.80	77.19	13.0

M11

Event#: 1 MS(E+) Ret. Time : 43.445 Scan# : 5038

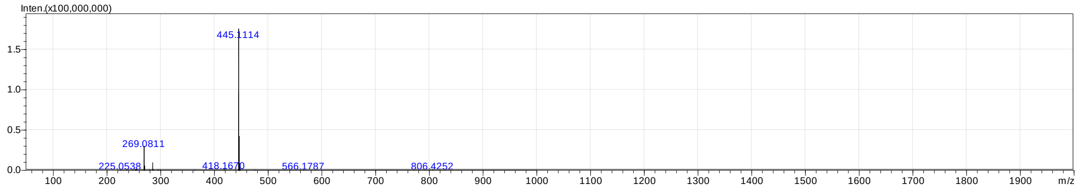

Event#: 4 MS(E-) Ret. Time : 43.445 Scan# : 5041

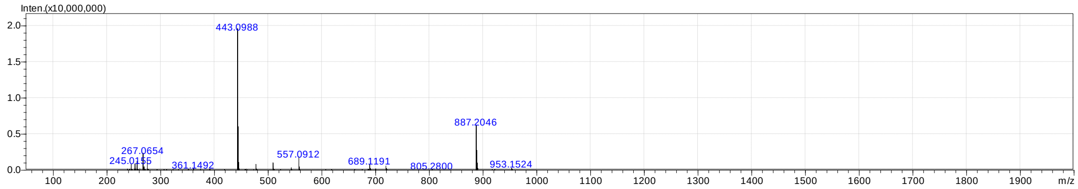

Rank	Score	Formula (M)Ion		Meas. m/z	Pred. m/z	Diff (mDa)	Diff (ppm)	Iso Score	DBE
1	69.85	C22 H20 O10	[M+H]+	445.1114	445.1135	-2.1	-4.72	77.02	13.0

M12

Event#: 1 MS(E+) Ret. Time : 43.703 Scan# : 5068

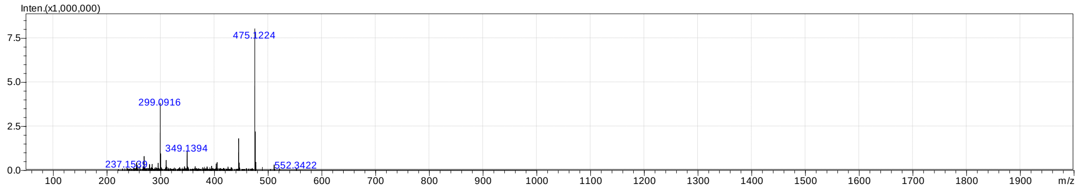

Event#: 4 MS(E-) Ret. Time : 43.703 Scan# : 5071

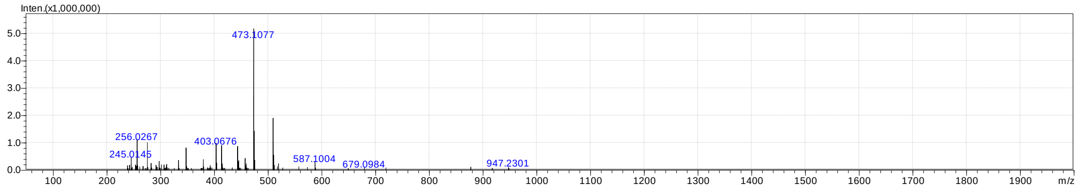

Rank	Score	Formula (M)Ion		Meas. m/z	Pred. m/z	Diff (mDa)	Diff (ppm)	Iso Score	DBE
1	77.12	C23 H22 O11	[M+H]+	475.1224	475.1240	-1.6	-3.37	81.98	13.0

M13

Event#: 1 MS(E+) Ret. Time : 44.168 Scan# : 5122

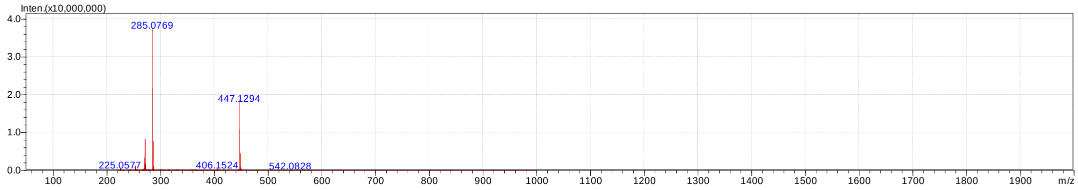

Event#: 4 MS(E-) Ret. Time : 44.168 Scan# : 5125

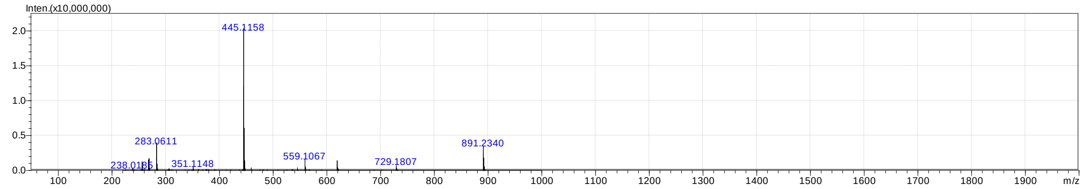

Rank	Score	Formula	(M)Ion	Meas. m/z	Pred. m/z	Diff (mDa)	Diff (ppm)	Iso Score	DBE
1	99.01	C22 H22 O10	[M+H]+	447.1294	447.1291	0.3	0.67	99.01	12.0

M14

Event#: 1 MS(E+) Ret. Time : 44.988 Scan# : 5217

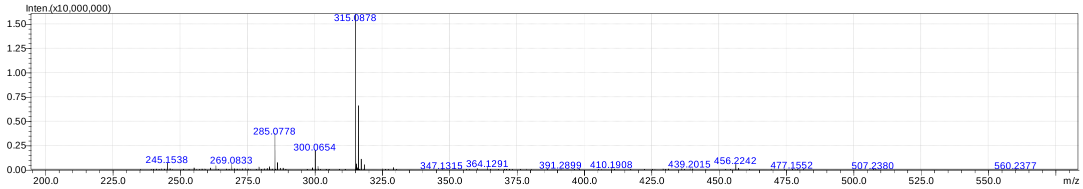

Event#: 4 MS(E-) Ret. Time : 44.988 Scan# : 5220

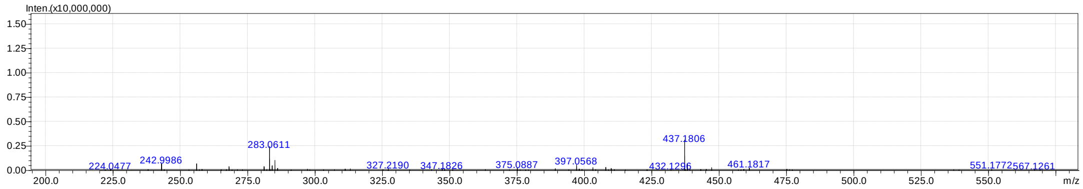

Rank	Score	Formula	(M)Ion	Meas. m/z	Pred. m/z	Diff (mDa)	Diff (ppm)	Iso Score	DBE
1	53.87	C16 H12 O5	[M+H]+	285.0778	285.0763	1.5	5.26	61.63	11.0

M15

Event#: 1 MS(E+) Ret. Time : 45.652 Scan# : 5294

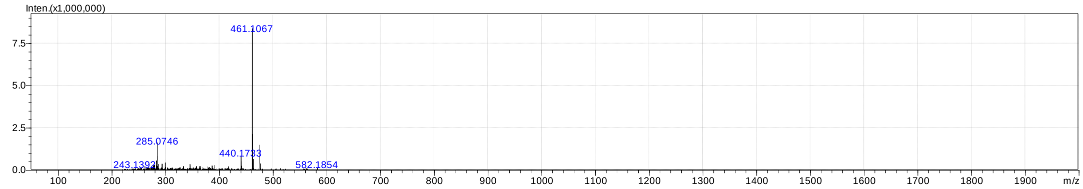

Event#: 4 MS(E-) Ret. Time : 45.652 Scan# : 5297

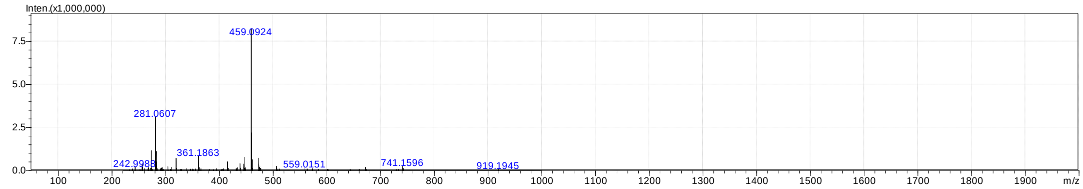

Rank	Score	Formula	(M)Ion	Meas. m/z	Pred. m/z	Diff (mDa)	Diff (ppm)	Iso Score	DBE
1	83.77	C22 H20 O11	[M-H]-	459.0924	459.0927	-0.3	-0.65	83.77	13.0

M16

Event#: 1 MS(E+) Ret. Time : 48.427 Scan# : 5616

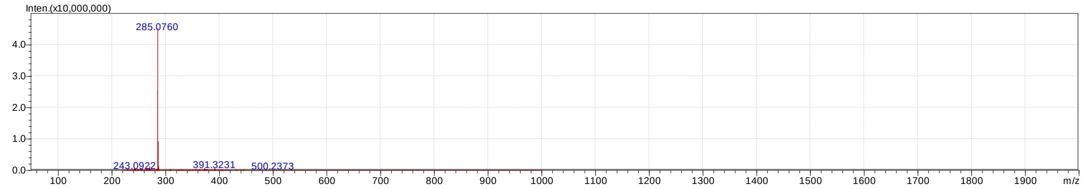

Event#: 4 MS(E-) Ret. Time : 48.427 Scan# : 5619

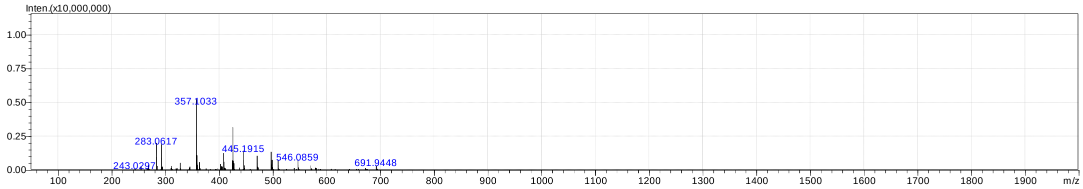

Rank	Score	Formula (M)Ion	Meas. m/z	Pred. m/z	Diff (mDa)	Diff (ppm)	Iso Score	DBE
1	85.95	$C_{16}H_{12}O_5$ [M+H]+	285.0760	285.0763	-0.3	-1.05	86.06	11.0

M17

Event#: 1 MS(E+) Ret. Time : 48.935 Scan# : 5675

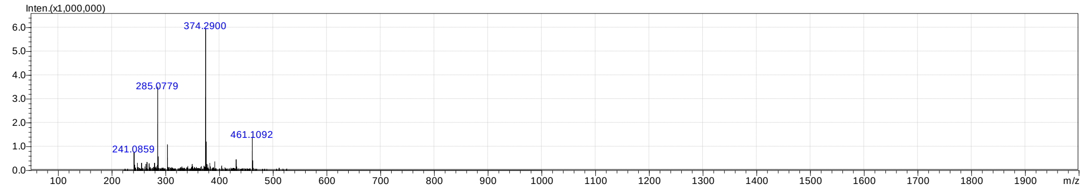

Rank	Score	Formula (M)Ion	Meas. m/z	Pred. m/z	Diff (mDa)	Diff (ppm)	Iso Score	DBE
1	69.13	$C_{16}H_{12}O_5$ [M+H]+	285.0779	285.0763	1.6	5.61	82.39	11.0

M18

Event#: 1 MS(E+) Ret. Time : 53.890 Scan# : 6250

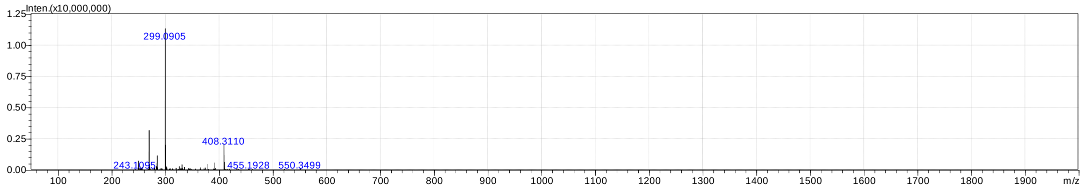

Rank	Score	Formula (M)Ion	Meas. m/z	Pred. m/z	Diff (mDa)	Diff (ppm)	Iso Score	DBE
1	64.81	$C_{17}H_{14}O_5$ [M+H]+	299.0905	299.0919	-1.4	-4.68	71.38	11.0

M19

Event#: 1 MS(E+) Ret. Time : 67.932 Scan# : 7877

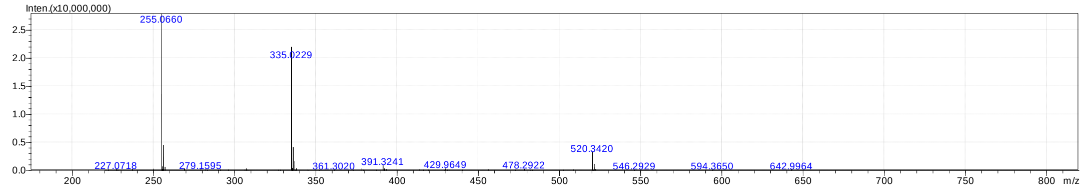

Event#: 4 MS(E-) Ret. Time : 67.932 Scan# : 7879

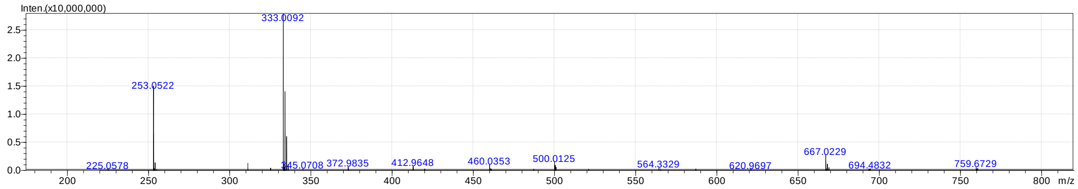

Rank Score Formula (M)Ion Meas. m/z Pred. m/z Diff (mDa) Diff (ppm) Iso Score DBE

1 83.32 C15 H10 O7 S [M+H]+ 335.0229 335.0225 0.4 1.19 83.72 11.0

M20

Event#: 1 MS(E+) Ret. Time : 76.412 Scan# : 8861

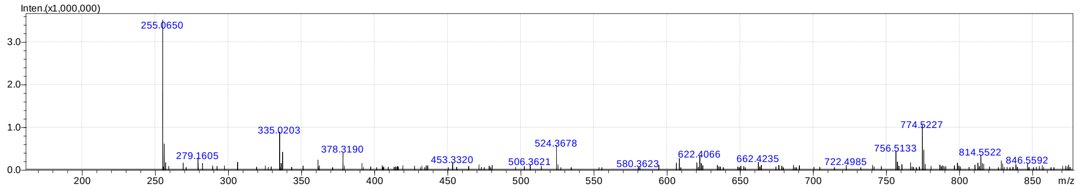

Event#: 4 MS(E-) Ret. Time : 76.412 Scan# : 8864

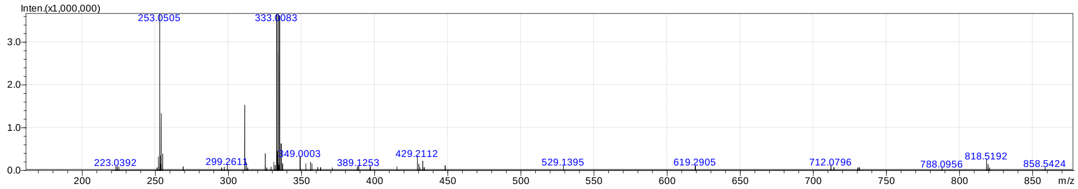

Rank Score Formula (M)Ion Meas. m/z Pred. m/z Diff (mDa) Diff (ppm) Iso Score DBE

1 45.22 C15 H10 O7 S [M-H]- 333.0083 333.0069 1.4 4.20 49.15 11.0

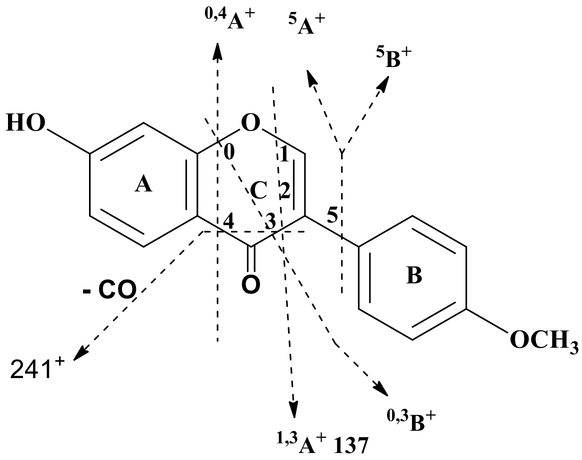

Figure S23. Nomenclature adopted for cross-ring cleavages of formononetin.

Table S1. Proposed metabolites of formononetin in hepatic S9 incubated sample by LC/MS analysis.

metabolites	t_R (min)	$[M+H]^+$ (m/z)	$[M-H]^-$ (m/z)	Main fragment Ion MS² MS³
Sm0(formononetin)	31.205	269.0852	267.0669	269,237,213,163,118,107
Sm1	18.568	447.1289	445.1134	447,285(162),229,152
Sm2	19.663	271.0597	269.0452	271,253,225,215,197,169,153,141
Sm3	20.413		445.1115	
Sm4	20.897	431.1337		431,269(162),237,213,181,152

Sm5	21.483	301.0699	299.0560	301,286,269,241,229,153
Sm6	22.458	417.1179	415.1011	417,285(-132),268,152,124
Sm7	22.847	255.0659	253.0493	255,227,199,152,137,109
Sm8	23.227	417.1188	415.1025	447,285(-162),253,152,123
Sm9	23.425	271.0606		271,253,225,215,197,169,153
Sm10	24.080	285.0755	283.0599	285,253,225,197,141
Sm11	25.132	417.1193	415.1055	417,285(-132),253,152,123
Sm12	27.053	285.0811	283.0611	285,211,183,152
Sm13	27.518	285.0756	283.0604	285,270,229,211,197,183,152
Sm14	32.385		299.385	299,270,254,237,213,181

Table S2. Proposed metabolites of ononin in rat biological sample by LC/MS analysis.

metabolites	t_R (min)	$[M+H]^+$ (m/z)	$[M-H]^-$ (m/z)	Main fragment Ion MS^2 MS^3
M1	33.707	431.0954	429.0815	429,253(-176),224,208,175,135
M2	34.397		431.0982	431,255(-176),175,149
M3	35.872		429.0833	429,253(-176),224,175
M4	36.372		431.0967	
M5	38.525		459.0938	459,283(-176),268
M6	39.447		417.1188	
M7	39.705		417.1181	
M8	42.007	461.1097	459.0923	461,285(-176),270,152,123
M9	42.627	255.0668	253.0525	255,199,152,137
M10	43.143	445.1127	443.0983	445,269(-176),237,118
M11	43.445	445.1114	443.0988	443,267(-176),252,175
M12	43.703	475.1224	473.1077	
M13	44.168	447.1294	445.1158	445,269,254,175,135
M14	44.988	285.0778	283.0611	283,268,224,131
M15	45.652	461.1067	459.0924	461,285(-176)
M16	48.427	285.0760	283.0617	283,268,224
M17	48.935	285.0779		285,241
M18	76.412	335.0203	333.0083	333,253(-80),224,135
M19	53.890		299.0905	299,284,243,166,137
M20	67.932	335.0229	333.0092	333,253(-80),225,211,135
M21	52.977	269.0847	267.0654	269,237,181,152,118
